# Central Gain Control in Tinnitus and Hyperacusis

**DOI:** 10.3389/fneur.2014.00206

**Published:** 2014-10-24

**Authors:** Benjamin D. Auerbach, Paulo V. Rodrigues, Richard J. Salvi

**Affiliations:** ^1^Department of Communicative Disorders and Sciences, Center for Hearing and Deafness, University at Buffalo, The State University of New York, Buffalo, NY, USA

**Keywords:** tinnitus, hyperacusis, central gain enhancement, lateral inhibition, homeostatic plasticity

## Abstract

Sensorineural hearing loss induced by noise or ototoxic drug exposure reduces the neural activity transmitted from the cochlea to the central auditory system. Despite a reduced cochlear output, neural activity from more central auditory structures is paradoxically enhanced at suprathreshold intensities. This compensatory increase in the central auditory activity in response to the loss of sensory input is referred to as central gain enhancement. Enhanced central gain is hypothesized to be a potential mechanism that gives rise to hyperacusis and tinnitus, two debilitating auditory perceptual disorders that afflict millions of individuals. This review will examine the evidence for gain enhancement in the central auditory system in response to cochlear damage. Further, it will address the potential cellular and molecular mechanisms underlying this enhancement and discuss the contribution of central gain enhancement to tinnitus and hyperacusis. Current evidence suggests that multiple mechanisms with distinct temporal and spectral profiles are likely to contribute to central gain enhancement. Dissecting the contributions of these different mechanisms at different levels of the central auditory system is essential for elucidating the role of central gain enhancement in tinnitus and hyperacusis and, most importantly, the development of novel treatments for these disorders.

## Introduction

Sensorineural hearing loss due to noise-exposure, aging, ototoxic drugs, or ear diseases that damage the sensory hair cells and/or auditory neurons in the cochlea is a significant sensory deficit that dramatically and negatively affects an individual’s quality of life and social interactions (1). Hearing loss often gives rise to subjective tinnitus, a phantom ringing, buzzing, or hissing sensation that occurs in the absence of an external sound, and hyperacusis, an auditory hypersensitivity disorder in which low- to moderate-intensity sounds are perceived as intolerably loud or even painful (2–4). Approximately 50 million Americans report experiencing tinnitus, 16 million of which report the experience as persistent (5). Although the prevalence of hyperacusis is slightly less than tinnitus (6), its occurrence is likely underestimated because many tinnitus patients are unaware of their reduced sound tolerance (7). The social and economic burden of tinnitus and hyperacusis is substantial. Tinnitus ranks number one among disability payments by the Veterans Administration in 2009, costing a total of $1.1 billion (8). Despite the high prevalence, significant economic impact, and immense impairment on quality of life, there are currently no FDA approved drugs to treat tinnitus or hyperacusis.

Development of pharmacotherapy for tinnitus and hyperacusis has been hindered by a poor understanding of the underlying pathophysiology of these disorders. Peripheral auditory damage has consistently been identified as the primary risk factor for both tinnitus and hyperacusis (3). Recent studies have demonstrated that even tinnitus patients with “normal” clinical audiograms are likely to have some subtle hearing disruptions that are likely triggers for tinnitus and hyperacusis (7, 9, 10). Much like phantom limb pain, tinnitus is often perceived as originating in the damaged ear and therefore early models of tinnitus proposed that the phantom auditory sensation was a consequence of a pathological increase in spontaneous neural activity in the peripheral sensory receptors or auditory nerve (AN) (11–14). However, models of tinnitus based solely on spontaneous hyperactivity in the cochlea are difficult to reconcile with several experimental findings. For example, physiological studies found a lack of change or a reduction in spontaneous AN activity after damage to outer hair cells (OHCs) or inner hair cells (IHCs), not an increase (15). Moreover, transection of the AN, which leads to a complete disruption of neuronal activity from the cochlea, fails to consistently abolish tinnitus, and in some cases, even exacerbates its perception (16). Finally, the psychophysical masking of tinnitus is incompatible with a cochlear origin; for example, low-level sounds presented to the contralateral ear are often capable of masking tinnitus perceived in the ipsilateral ear (17). Hyperacusis and loudness recruitment are also difficult to explain by peripheral pathophysiology, as the perception of loudness increases at an abnormally rapid rate despite the fact that cochlear output is substantially reduced in a damaged ear (4). These observations, along with more recent imaging studies, suggest that tinnitus and hyperacusis, while triggered by cochlear damage, result from a maladaptation of the central auditory system to this peripheral dysfunction, similar to phantom limb pain (18, 19).

**Figure 1:**
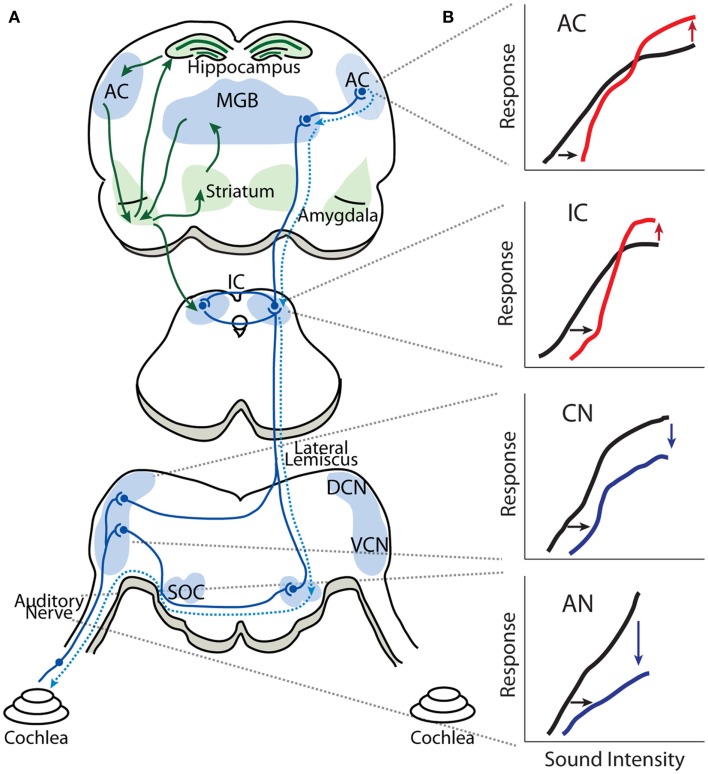
**Gain enhancement in the central auditory system**. **(A)** Schematic showing the general anatomical organization of the auditory system. The nuclei and areas of the auditory system are highlighted in blue. The ascending anatomical projections are depicted with solid blue lines whereas the dotted blue lines represent descending projections. Limbic regions that respond to auditory stimuli and display some evidence of central gain enhancement are highlighted in green. **(B)** Schematics of intensity-level functions collected from the auditory nerve (AN), cochlear nucleus (CN), inferior colliculus (IC), and auditory cortex (AC). The black lines represent baseline intensity-level functions. Cochlear damage via noise or ototoxic drug exposure results in depression of sound-evoked responses in lower auditory structures (blue lines) but results in enhancement of suprathreshold responses in higher areas (red lines), despite thresholds being shifted (black arrows). SOC, superior olivary complex; VCN, ventral cochlear nucleus; DCN, dorsal cochlear nucleus; IC, inferior colliculus; MGB, medial geniculate body; AC, auditory cortex.

While the central origin of tinnitus and hyperacusis is now widely recognized, there is no broad consensus as to the specific mechanisms or loci generating these hearing disorders. Several neurophysiological models of tinnitus have been proposed, including tonotopic expansion/reorganization (19, 20), enhanced neural synchrony (21–23), increased spontaneous hyperactivity (24, 25), and aberrant filtering of auditory information by limbic regions (26–28). Here, we will review evidence for the Central Gain Model of tinnitus and hyperacusis. While hearing loss induced by noise-exposure or ototoxic drugs reduces the neural activity transmitted from the cochlea to the central auditory system, spontaneous and sound-evoked responses at higher auditory structures, such as the auditory cortex (AC), medial geniculate body (MGB), and inferior colliculus (IC), are paradoxically increased (Figure 1) (29–35). This observed increase in neural activity is at the core of the Central Gain Model, which proposes that tinnitus and hyperacusis result from a compensatory increase in gain or neural amplification in the central auditory system to compensate for a loss of sensory input from the cochlea. To put the Central Gain Model into perspective, we will first review the general organization of the auditory system and then examine the experimental evidence for central gain enhancement, focusing on where, when, and how it is triggered. Next, we will consider potential cellular and molecular mechanisms of central gain enhancement and how the loss of auditory input may initiate these changes. Finally, we will discuss how central gain enhancement may contribute to the generation and maintenance of tinnitus and hyperacusis, and consider potential research strategies to address remaining issues regarding the role of auditory gain enhancement in tinnitus and hyperacusis.

## General Organization of the Auditory System

A remarkable feature of the auditory system is that a complex sound can be separated into its constituent frequencies by the hydromechanical properties of the basilar membrane located in the organ of Corti in the cochlea. High-frequency components of complex sounds preferentially stimulate IHCs near the basal end whereas low-frequencies preferentially excite IHCs near the apical end of the basilar membrane. Each of the ~3,500 IHCs is innervated by 10–30 unbranched afferent fibers from spiral ganglion neurons with each afferent fiber forming a single-bouton-like ending on an IHC (36–39). Therefore, the output of each auditory fiber provides information from a restricted region of the cochlea. This cochlear frequency-place map (tonotopic organization) is maintained throughout the central auditory system.

Figure 1 is a highly simplified schematic showing the general anatomic-physiological organization of the auditory system. Neural activity transmitted by the AN (VIII cranial nerve) enters the brainstem; each AN fiber branches into an ascending branch terminating in the antero-ventral cochlear nucleus (AVCN), and a descending branch terminating in the dorsal cochlear nucleus (DCN) and posteroventral cochlear nucleus (PVCN). The VCN sends processed auditory information to the ipsilateral lateral lemniscus (intermediary, ventral and dorsal lateral lemniscus nuclei) as well as to ipsilateral and contralateral superior olivary complex (lateral superior olive, medial superior olive, and medial nucleus of the trapezoid body). The DCN sends processed auditory information directly to the contralateral IC in the midbrain, which also receives auditory information from the contralateral and ipsilateral superior olivary complex as well as from the ipsilateral lateral lemniscus. Thus, the IC is an auditory information processing hub that receives and integrates auditory information from several auditory nuclei of the brainstem. The fibers from the IC project ipsilaterally and contralaterally to the MGB of the thalamus, which is subdivided in to ventral, dorsal, and medial MGB, where the ventral MGB transmits auditory signals to the primary AC. The AC is subdivided into several areas in mammalians, which have reciprocal projections to areas of the prefrontal cortex (40–42). Important for our discussion is the fact that sound processing activates several limbic structures as well, such as the amygdala, striatum, and hippocampus, through projections from the AC and MGB (43–45). It has recently been proposed that limbic structures are essential in enabling tinnitus distress (26, 46, 47) and it has been found that sound-evoked responses in limbic regions are enhanced in animal models of tinnitus (31, 35).

## Gain Enhancement in the Central Auditory System

**Figure 2:**
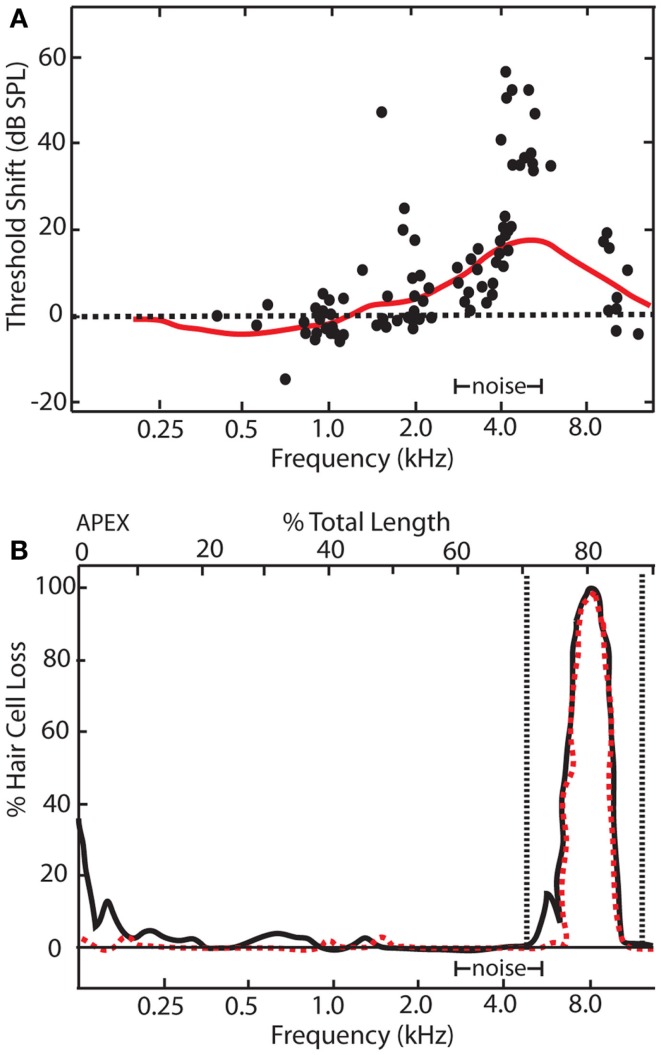
**Relationship between electrophysiological and behavioral threshold shifts**. **(A)** Schematic showing temporary threshold shifts in adult chinchillas that were exposed to an octave band noise centered at 4 kHz with 86 dB SPL amplitude for 5 days. Electrophysiological recordings of auditory nerve fibers revealed thresholds shifts of up to 70 dB SPL at characteristic frequencies between 4 and 11 kHz (black dots). However, behavioral audiograms (red line) revealed relatively smaller behavioral threshold shifts in comparison, ranging from 5 to 20 dBs SPL at frequencies between 4 and 11 kHz. **(B)** Cochleogram showing narrow lesions of inner (dotted red line) and outer hair cells (black line) over the 1 mm that correlated with the frequency of the electrophysiological and behavioral threshold shifts [modified from Ref. (48)].

The observation of enhanced sound-evoked neural responses after auditory damage is by no means a recent phenomenon. Some of the earliest hints for central gain enhancement came from studies examining the relationship between auditory threshold shifts assayed electrophysiologically and behaviorally in noise-exposed animals (48, 49). Interestingly, while noise-exposure caused a substantial threshold shift at the AN measured electrophysiologically, behavioral audiograms revealed threshold shifts that were substantially smaller (Figure 2). It should be noted that electrophysiologically and behaviorally measured thresholds are likely to have different sensitivity to changes in auditory input, as behaviorally measured responses reflect a much larger spatial and temporal integration of information than most electrophysiologically measured events. However, these results suggest there may be compensatory neuronal mechanisms (e.g., off-frequency listening, central gain, or tonotopic map reorganization) to account for the loss of auditory input to the central auditory system. Indeed, studies on audiogenic seizure susceptibility in mice were some of the first to demonstrate that the central auditory system adapts to intense noise-exposures by enhancing neuronal activity in response to sound (50, 51).

More direct evidence for central gain enhancement comes from studies examining evoked-potentials elicited by electrical stimulation (ES) of central auditory structures, thereby completely bypassing peripheral input, before and after noise-exposure (52–54). These studies consistently found lower thresholds and increased amplitudes for ES-evoked responses in deafened animals, suggesting that the central auditory system had become hyper-responsive as a result of deafening. Similar to the observed enhancement of central auditory evoked-responses, behavioral threshold detection of ES of various auditory nuclei was also enhanced after sensorineural hearing loss, demonstrating a direct relationship between central gain enhancement and auditory perception (52, 53). Numerous studies have now shown that neuronal enhancement occurs across several species of mammals at different ages, including adulthood, and it is clear that this enhancement does not originate at the sensory periphery (29, 49–51, 52, 55–59). While these early studies are illuminating, several questions remain. First, while central gain enhancement has been observed in several auditory areas, it is unclear where this hyperactivity is initiated, how it is transmitted between regions, and what is the relative contribution of each area to the overall changes in activity. Further, it is unclear if this neuronal enhancement is restricted to specific tonotopic regions corresponding to the damaged cochlear region or if it is more widespread. Below we will review the origins, temporal dynamics, and spectral profile of central gain enhancement.

### Origins of central gain enhancement

**Figure 3:**
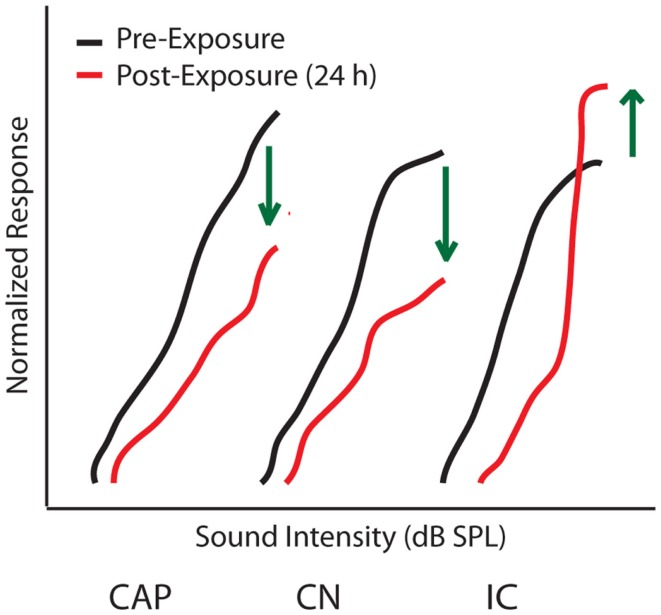
**Origins of central gain enhancement**. Schematized data for amplitude-level functions to a 1 kHz tone chronically recorded from chinchillas at the round window (CAP), cochlear nucleus (CN), and inferior colliculus (IC), before (black lines) and 24 h after (red lines) noise-exposure of 105 dB SPL at 2.8 kHz for 2 h. Green arrows indicate the direction of amplitude change after noise-exposure. Responses are normalized to maximum response before the noise-exposure [modified from Ref. (57)].

Determining the origins of central gain enhancement is complicated by the complex, interconnected nature of the auditory system (Figure 1). To identify potential sites where noise-induced hyperactivity might originate, chronic electrodes were implanted on the round window, in the cochlear nucleus (CN), and in the IC to record local field potential (LFP) input–output functions from awake chinchillas before, 24 h, and 30 days after they were exposed to a 105 dB SPL, 2.8 kHz tone for 2 h (29, 57). This noise-exposure caused a large reduction in the compound action potential (CAP) amplitude-level functions recorded from the round window as well a similar reduction in the LFP from the CN (Figure 3). In the IC however, while the threshold was shifted, the amplitude-level function increased significantly once the threshold was crossed, becoming substantially larger than those seen before sound exposure (Figure 3) (57, 60). Thirty days post-noise-exposure, responses from the IC showed substantial recovery in the threshold shifts but remarkably continued to displayed marked enhancement at high intensities. Other investigators, using similar chronic recordings techniques in guinea pigs, demonstrated that exposure to broadband white noise (120 dB SPL, 1 h) also decreased CAP amplitude-level functions while enhancing those in the IC and AC at suprathreshold intensities (55). Interestingly, this study found that neuronal enhancement in the AC was more rapid and robust than in the IC (55). Thus, these results indicate that both the induction and maintenance of central neuronal enhancement is clearly seen at the level of the IC after permanent threshold shifts (PTS) (57, 60).

The above studies indicate that central gain enhancement may originate at the level of the IC. However, while LFP recordings from the CN failed to show obvious evidence of neuronal enhancement post-noise-exposure (52, 56, 57), it is still possible that the neuronal enhancement seen in the IC and above could have some of its origins between the IC and CN. The LFP is believed to be summed excitatory post-synaptic potentials (EPSPs) of hundreds of neurons around the electrode tip (61–63), and thus reflects responses from a large, synchronous neuronal population. Therefore, if central gain enhancement is restricted in a cell-type specific or localized manner, it would be difficult to measure such an effect with global LFP amplitude-level functions. Furthermore, enhanced LFP amplitudes may not only be due to increased post-synaptic strength but could also be due in part to increased neural synchrony or enhanced pre-synaptic transmission. Thus, mechanistically, it is unclear if increased neuronal activity in the IC is the result of changes within the IC itself or to increased output from more peripheral ascending projecting neurons (Figure 1).

Single-unit recordings in the VCN and DCN have revealed there is indeed enhancement of sound-evoked responses in these areas, but in a cell-type restricted manner (49, 64). In the VCN, brief noise-exposure (105–115 dB SPL, 5 min) resulted in depressed rate-level functions in most recorded neurons, consistent with earlier chronic LFP recordings. However, rapid neuronal enhancement was observed in primary-like-notch cells. It is unclear how this enhancement contributes to increased LFP amplitude in the IC because of the complex pattern of anatomical projections from the VCN (65). The only cells that have a direct projection to the IC from the VCN are stellate cells (chopper type firing pattern cells). Interestingly, a more recent study found that noise-induced hearing loss in cats caused neuronal enhancement specifically in chopper cells in the VCN, thereby suggesting that amplitude-level enhancement found after hearing loss in the IC could be explained by alterations in the VCN (66). This study also found that, contrary to previous results, primary-like and primary-like-notch neurons show a depression of their rate-level function instead of an enhancement. However, these recordings examined long-term changes (37–130 days after noise-exposures) as opposed to the immediate changes examined in previous studies (64). Single-unit recordings in the DCN show that noise-induced hearing loss also significantly enhanced the rate-level function of buildup type neurons (putative fusiform cells) in guinea pigs (67) and chinchillas (68). Similar to chopper cells in the VCN, the neuronal enhancement seen in the DCN was detected 3–5 months after sound exposure, arguing that gain enhancement was a long-term adaptation to the noise-induced cochlear hearing loss (67, 68). Interestingly, neuronal enhancement in the DCN was more prominent in animals that had developed tinnitus (67), suggesting a correlation between dynamic range disruption and tinnitus perception.

Taken together, these single-unit recordings from the DCN and VCN suggest that enhanced LFP amplitude-level functions in the IC after long-term noise-exposures are at least in part transmitted from the CN. However, single-unit recordings from the IC demonstrate that there is likely further neuronal enhancement at the level of the IC. The rate-level function of VCN neurons displayed enhancement on the order of 25% after noise-exposures (64), whereas enhancement of rate-level functions in neurons of IC were on the order of 40–50% (69–71). Moreover, a larger proportion of IC neurons (70%) showed a significant increase in rate-level functions compared to those in the VCN (23%). Thus, while gain enhancement can be observed at the earliest levels of the central auditory system, it is likely modified at multiple levels and not passively transmitted between regions.

### Temporal dynamics of central gain enhancement

**Figure 4:**
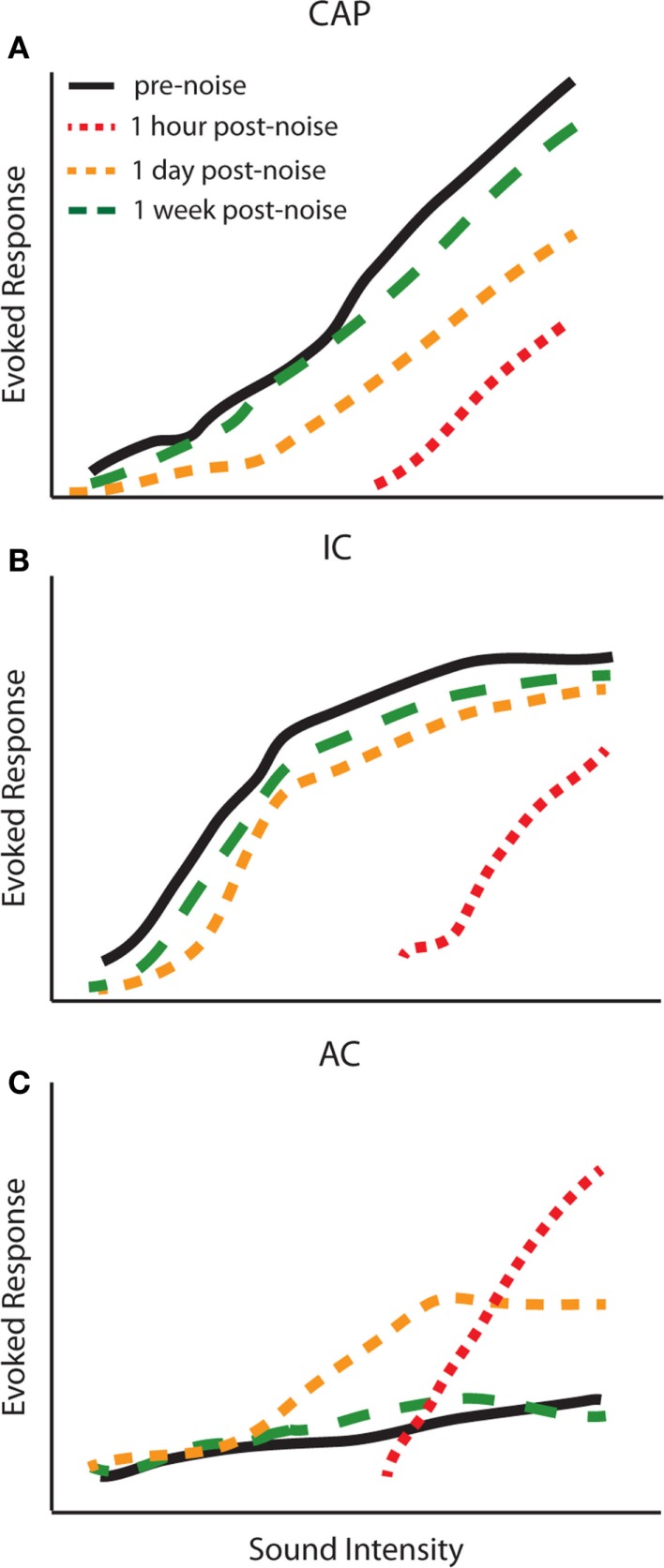
**Temporal dynamics of central gain enhancement**. Schematized data representing the temporal dynamics of noise-induced changes to amplitude-level functions in response to click stimuli from the **(A)** compound action potential (CAP), **(B)** inferior colliculus (IC), and **(C)** auditory cortex (AC). The amplitude-level functions were computed from chronic recordings of CAP and LFPs from the IC and AC before (black lines), 1 h (red lines), 1 day (yellow lines), and 1 week (green lines) post-noise-exposure. The parameters for noise-exposure were white broadband noise at 115 dB SPL for 1 h [modified from Ref. (72)].

The studies above suggest that changes in central gain levels are dynamic, as the magnitude and even the cell-types displaying central gain enhancement vary over time post-exposure. It is therefore important to determine when these changes occur in different auditory structures to better understand the origins of gain enhancement. Interestingly, the temporal profile of central gain changes suggests that enhancement may not simply be transmitted from lower auditory structures to higher ones. For instance, simultaneously measured amplitude-level functions from the CAP, IC, and AC before and up to 2 weeks post broadband white noise-exposure (120 dB SPL, 1 h) demonstrated a complex temporal profile of auditory gain enhancement (72) (Figure 4). This study found that amplitude-level functions from the AN and IC were both reduced 1 h post-noise-exposure (Figures 4A,B; red line). Surprisingly, while the threshold was shifted in the AC, suprathreshold responses were greatly enhanced 1 h post-exposure (Figure 4C; red line). The CAP amplitude remained depressed for several days, yet responses were still enhanced in the AC and were back to normal in the IC at 24 h (Figure 4, yellow lines). Importantly, although there was never a full recovery to the pre-noise-exposure levels at the AN, the amplitude-level functions from the IC and AC displayed similar amplitudes as before noise-exposure (Figure 4, green lines). Similar results have now been reported across several species as well as with multi-unit rate-level functions (30, 32, 55, 57, 72). Thus, there is a general agreement that while gain enhancement is seen in both the IC and AC, the dynamics of the neuronal enhancement are very different in these structures. The AC generally displays a fast enhancement after noise-exposure whereas it seems that this enhancement manifest itself at a later time in the IC (Figure 4) (30, 32, 55, 59, 72–74).

The difference in temporal dynamics between the AC and IC could be due to intrinsic properties that allow the AC to display a faster adaption than the IC to the lack of peripheral sensory input. Alternatively, it could be due to the complexity of changes in the IC and its varied ascending projections. For instance, both enhancements and depression of rate-level functions are seen in different subclasses of neurons in the IC (75). It is important to bear in mind that approximately one-third of the feed-forward monosynaptic connections from the IC to the MGB, the main auditory input into A1, are constituted by GABAergic projections (76–79). Due to this mix of excitatory and inhibitory projections, neuronal enhancement or depression in the IC can either induce or inhibit gain control in the AC. More studies examining connectivity between the IC, MGB, and AC after noise-damage are needed to determine how gain enhancement may be transmitted between these regions. Another intriguing possibility is that changes may occur initially in the AC and are then transmitted to the IC via descending projections (Figure 1). There is extensive evidence to suggest that descending corticofugal projections are not only involved in short-term modulation of auditory processing but long-term changes as well, and have specifically been implicated in gain control (80–83). Indeed, inactivation of the AC has been shown to modulate firing rates and rate-level functions in the IC in a complex manner (75). Further experiments are required to determine if disrupting central gain changes in the AC can prevent enhancement in lower structures.

It is important to keep in mind that there are several variables that can contribute to the temporal profile of gain enhancement. It seems that the type and level of noise-trauma greatly influences the temporal dynamics of auditory gain enhancement. In the above studies that observed concomitant enhancement in the AC and depressed amplitude-level functions in IC, the noise-exposure used resulted in moderate threshold shifts (Figure 4) (32, 55, 72, 73, 74). However with more intense or prolonged noise-exposure, responses in the AC are decreased immediately after exposure as well, and then gradually increase over several hours to days (73, 74). Indeed, it has been shown that the timing of neuronal enhancement in the AC varies systematically with respect to the amount of temporary threshold shift (TTS) (72). For instance, high intensity noise-exposure (125 dB SPL, 30 min) causing a TTS of 50–60 dB resulted in peak neuronal enhancement 6–8 h post-noise-exposure, whereas for a low intensity noise-exposure (105–115 dB SPL, 1 h) causing a TTS of 5–40 dB, the highest peak of neuronal enhancement occurred 1 h post-exposure (72, 73).

There is considerable variability in the temporal dynamics of gain enhancement across animals in response to similar noise-exposure as well (73, 74). Rats exposed to three types of noises for 1 h (broadband, 0.8–20 kHz, 105–120 dB SPL; high-frequency narrowband noise, 12.7–20 kHz, 105–120 dB SPL; and low frequency narrowband noise, 0.1–7 kHz, 105–120 dB SPL) show variable degrees of neuronal enhancement and threshold shifts (73). While most animals have a depressed response at 1 h, some animals exhibited peak neuronal enhancement at 1 day post-exposure, while in others peak enhancement was observed 3 days to 1 week post-noise-exposure (72, 73). Thus, the time-course of the neuronal enhancement across animals as well as across structures is variable (30, 32, 55, 72, 74, 84). Interestingly, it has been shown that the original amplitude of the evoked-responses before noise-exposure correlates with the amount of neuronal enhancement seen, such that small evoked-responses are correlated with larger neuronal enhancement (84). This suggests that intrinsic properties of the AC across animals are important for determining the ability of this structure to compensate for lost sensorineural inputs.

### Spectral profile of gain enhancement

**Figure 5:**
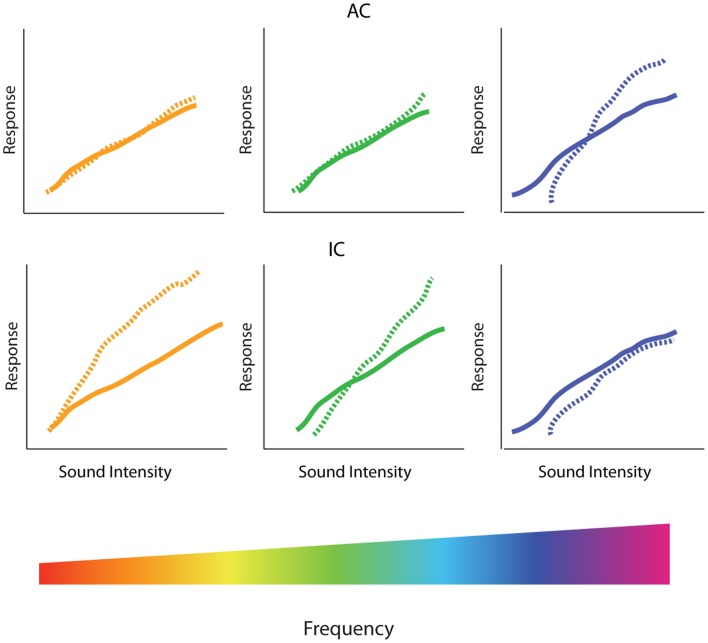
**Spectral profile of central gain enhancement**. Schematized data representing the amplitude-level functions before (solid lines) and after (dotted lines) restricted high-frequency hearing loss in the inferior colliculus (IC) and auditory cortex (AC). (Bottom) The tonotopic organization of the cochlea is depicted in the rainbow color spectrum with orange colors of the spectrum representing low-frequencies, green colors representing middle frequencies, and blue colors representing high-frequencies. (Middle) In the IC, high-frequency hearing loss results in increased threshold and depressed amplitude-level functions in the tonotopic region corresponding to the region of hearing loss (blue), while suprathreshold responses are enhanced at low and middle frequencies (orange and green, respectively). (Top) In contrast, amplitude-level functions from the AC are enhanced in the region of hearing loss (blue) while relative unchanged at low (orange) and middle (green) frequencies.

The tonotopic organization of the cochlea is maintained as information ascends through the auditory system, and alterations to this organization after hearing loss have been suggested to contribute to altered auditory processing, hearing disorders, and tinnitus (9, 85, 86). Thus, it is important to determine if central gain enhancement occurs in a frequency-dependent manner, as it will likely shed light on both the consequences and mechanisms of enhancement. Restricted hearing loss to specific frequencies allows for frequency-dependent examination of central gain enhancement. For instance, chinchillas exposed to a loud pure tone (2 kHz, 105 dB SPL, 5 days) showed a 40% loss of OHC around the 2–3 kHz cochlear region and a PTS of 20–30 dB between 2 and 8 kHz (56, 60). LFP recordings from the IC of these animals showed that low-frequencies lying below the edge of the hearing loss region were enhanced, whereas high-frequencies within the region of hearing loss were largely depressed (Figure 5, middle). Multi-unit recordings from the IC further confirm these results (34). Consequently, the neuronal enhancement of rate and amplitude-level functions in the IC show that not all tonotopic regions of the auditory pathway display gain control in the same manner.

As with temporal aspects of gain enhancement, there appears to be a dichotomy in the frequency dependency of gain changes in the AC verses the IC. While evidence suggests that gain enhancement is observed on the edge of the region of hearing loss in the IC, studies in the AC have observed the greatest enhancement within the region of hearing loss (32, 73, 74) (Figure 5, top). Other results, however, suggest that neuronal enhancement is triggered at low-frequencies immediately after the noise-exposure in the AC, whereas amplitude-level curves at frequencies within the region of cochlear trauma are first depressed after noise-exposure, and only show neuronal enhancement days after the exposure (72). Thus, the time-course of neuronal enhancement in the AC seems to vary in a frequency-dependent manner. This could potentially reflect a difference in mechanisms for gain enhancement observed within or outside the region of cochlear damage.

### Ototoxic drug-induced central gain enhancement

Noise-damage is the most common method used for inducing cochlear trauma (1). However, a number of ototoxic drugs cause cochlear damage as well and it is important to determine if central enhancement is generalizable to these types of cochlear trauma. Ototoxic drug exposure has several experimental benefits. For example, some drugs preferentially destroy specific structures in the cochlea and it is of interest to determine how specific types of cochlear lesions alter the gain of the central auditory system and how this relates to tinnitus or hyperacusis.

#### Carboplatin

While noise-exposure can cause trauma to both IHCs and OHCs, noise tends to preferentially damage OHCs (15, 87–89). This suggests that OHC loss could be the main trigger leading to enhanced neural activity in the central auditory system; indeed, some models suggest that OHC damage is essential for central gain enhancement (13, 14, 90). A critical test of this hypothesis requires inducing cochlear trauma that is restricted specifically to either OHCs or IHCs. Fortuitously, it is possible to induce selective IHC damage in chinchillas using carboplatin, a platinum-based, antineoplastic drug used to treat cancer, which is also known to cause tinnitus (71, 91–95).

**Figure 6:**
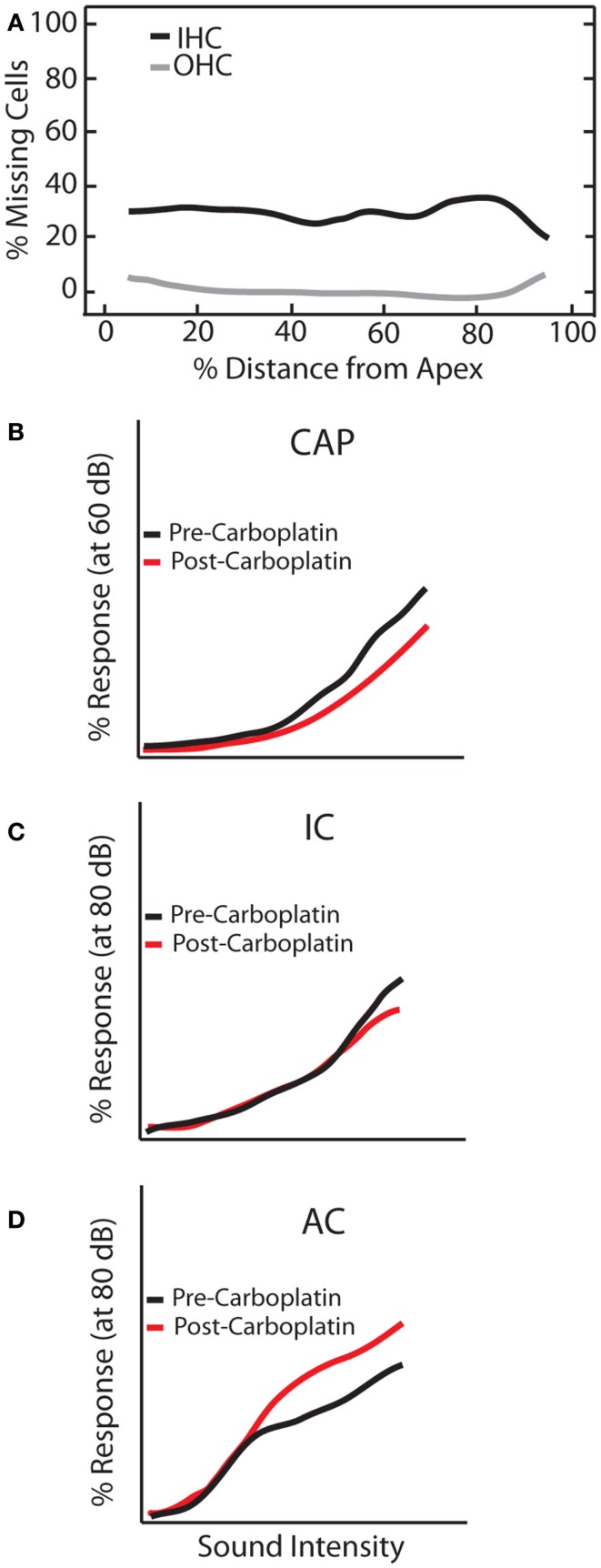
**Carboplatin-induced changes to peripheral and central auditory system**. Schematic representation of the effects of carboplatin treatment (30 mg/kg) on **(A)** hair cell loss, and amplitude-level functions from the **(B)** compound action potential (CAP), **(C)** inferior colliculus (IC), and **(D)** auditory cortex (AC). **(A)** Mean cochleogram showing the effects of carboplatin on OHC (gray lines) and IHC (black lines). While relatively little OHC loss was observed, an average of 30% of IHC are lost across the frequency-place map. **(B–D)** amplitude-level functions measured at 1 kHz before (black lines) and after (red lines) carboplatin treatment [modified from Ref. (102)].

Mild to moderate (30–60 mg/kg) doses of carboplatin selectively kills 20–40% of IHCs along the length of the cochlea while sparing OHCs (96–99) (Figure 6A). Accordingly, distortion product otoacoustic emissions (DPOAEs) remain functionally intact whereas the CAP is greatly reduced (94, 96, 100). However, the general physiological properties of the residual IHCs and auditory fibers seem to be largely unaffected as they retain sharp tuning curves and display relatively normal thresholds (94). Interestingly, despite normal neural and behavioral thresholds (101) substantial IHC loss resulted in a marked reduction in CAP amplitude but, remarkably, only a small reduction in the IC and robust neuronal enhancement in the AC (Figure 6). These effects were seen as early as 3 days post-carboplatin treatment, suggesting a fairly fast neuronal adaptation to the lack of sensory input provided by IHCs. Thus, these results show that neuronal enhancement is not solely triggered by damaging OHCs, but also by selective damage to IHCs. Interestingly, similar to noise-damage, there was much variability in gain enhancement observed between animals after carboplatin-induced IHC loss, even though the amount of IHC loss was relatively stable across animals (30–40%) (99, 102). This suggests that the variability in gain changes observed between animals is not strictly due to differences in peripheral damage but also depends on factors in the central auditory system.

#### Salicylate

Noise-damage is one of the most common risk factors for tinnitus and hyperacusis, however, the likelihood of developing these disorders after noise-induced hearing loss is still relatively low (3). On the other hand, high doses of sodium salicylate, the active ingredient in aspirin, not only causes cochlear hearing loss but also consistently induces tinnitus and hyperacusis in both humans and experimental animals (14, 35, 103–117). As such, salicylate administration is a useful experimental manipulation to infer whether the neuronal enhancement seen after noise-induced hearing loss is also consistently seen in animals that are experiencing tinnitus and hyperacusis. Furthermore, unlike most noise or ototoxic exposures, the effects of salicylate are rapid and reversible.

Experimental manipulations with sodium salicylate consistently reduce the sensory and neural output from the cochlea by disrupting both IHC (112, 114) and OHC function (108, 118–127). Consistent with noise-damage, systemic salicylate treatment also induces widespread gain enhancement in the central auditory system. Despite the fact that the neural output of the cochlea is greatly reduced by high dose salicylate, the suprathreshold LFP amplitudes in the IC are nearly the same as pre-salicylate amplitudes (116). These results imply that some gain enhancement has already taken place between the output of the AN and the IC. The MGB is considered an essential hub linking auditory perception and tinnitus distress (26, 128–130); however, its precise role in the generation of tinnitus and hyperacusis is poorly understood. Nevertheless, current-source density analysis (CSD) of the AC following salicylate treatment suggests that neural enhancement may be occurring in the MGB (131). CSD analysis revealed that the current sinks (net ionic flow corresponding to depolarization) in layer IV of the AC, which corresponds to monosynaptic inputs from the ventral MGB, were enhanced after salicylate treatment (61, 132–136). Indeed, LFP input–output functions recorded from the MGB confirmed that there is significant enhancement in this structure after systemic injection of salicylate (33, 114).

Several groups have found that salicylate significantly enhances suprathreshold LFP, multi-unit or single-unit discharges in the AC (110, 116, 137–139). Neuronal enhancement is apparent within the first hour post-salicylate (116, 138, 139). Thus, the time-course of enhancement induced by salicylate treatment and noise-induced TTS are similar. Importantly, CSD results show that longer latency supragranular and infragranular sinks, which likely reflect polysynaptic connections of horizontal fibers and intracortical or corticofugal neurons within A1 (61, 133–135, 140), are also enhanced by salicylate treatment (131). The neuronal enhancement of intracortical connections suggests that salicylate can directly impact neuronal processing in the AC. In agreement with this view, local application of salicylate to the AC enhanced sound-evoked amplitude-level functions in the AC without having any threshold shifts at low intensity stimulations (31, 114). In contrast, local application of salicylate to the round window caused a similar threshold shift as systemic administration, however, amplitude-level functions from the IC and AC were actually depressed rather than enhanced in this case (116). Taken together, these results suggest that the threshold shift following systemic salicylate treatment is cochlear in origin whereas the sound-evoked neuronal enhancement observed in the AC must be due to the direct effects of salicylate on the brain. The ability to dissociate peripheral hearing loss from central gain enhancement may indicate that salicylate is enhancing neural activity via a different mechanism than noise-exposure. However, the effect of sodium salicylate and noise-induced hearing loss have been directly compared in chronically implanted guinea pigs (74). Both noise and salicylate enhanced sound-evoked neural activity and increased the early peak of the LFP evoked-responses in AC in nearly identical ways. These results suggest that salicylate and noise-exposure may induce gain enhancement in a similar manner. A further test of this hypothesis would require determining the effects of salicylate administration on gain enhancement in animals previously exposed to noise.

### Central gain enhancement in limbic regions

Debilitating tinnitus and hyperacusis are often accompanied by negative emotions and symptoms such as anxiety, emotional distress, depression, sleep disturbances, and fear (141–143). The amygdala is a limbic structure that is involved in processing aversive auditory stimuli (144), has reciprocal connections to the MGB and AC (43–45), and is strongly implicated in tinnitus. Functional and structural MRI imaging studies of tinnitus patients have identified hyperactivity and morphological abnormalities in limbic regions involved in mood, memory, and motivation (26, 46, 47, 129, 130, 145–147). For instance, diffusion tensor imaging demonstrates that the white matter tract between the AC and amygdala are more strongly coupled in patients with tinnitus than controls (148). Furthermore, tinnitus is suppressed by infusing Amytal, a barbiturate sedative that enhances inhibition (149), into the artery providing blood flow to the amygdala, which decreases neuronal activity in this region (150). Thus, models that attempt to account for the strong negative affect associated with tinnitus or hyperacusis need to take in account abnormal neural activity in the limbic system. Indeed, high doses of salicylate that induce tinnitus, hyperacusis, and enhanced neural activity in the central auditory pathway also caused strong neural enhancement in the amygdala. Interestingly, systemic salicylate injection also caused some high- and some low-characteristic frequency (CF) neurons in the amygdala to shift their CFs to the mid-frequencies (8–16 kHz) resulting in an over representation of mid-CF neurons (35). This increase in the number of mid-CF neurons (10–20 kHz) closely matches the measured tinnitus pitch in rats given systemic salicylate (109, 110, 151, 152). The same up-shift and down-shift of low-CF and high-CF neurons has also been seen in the AC following systemic salicylate treatment (122). Moreover, when LFPs were recorded from the AC while applying salicylate locally to the amygdala, responses from the AC were greatly enhanced (31). These results show that gain control in the classical auditory system can be strongly modulated by the amygdala. Furthermore, these results suggest that limbic regions may play an important role in linking negative emotions to hyperacusis and tinnitus. Indeed, recent studies have demonstrated that salicylate treatment enhances auditory responses in several other limbic structures as well, including the striatum and hippocampus (35). Future studies are needed to determine if similar changes are observed with noise-damage.

## Mechanisms of Central Gain Enhancement

There is extensive *in vivo* evidence for central gain enhancement after cochlear damage. However, understanding how this enhancement manifests at the cellular-level is required not only to lend insight into the operation of the auditory system and abnormal auditory perception, but to provide valuable drug targets for the treatment of tinnitus and hyperacusis. Mechanistically, there are several synaptic or cellular alterations by which gain enhancement may be achieved: (1) a decrease in inhibitory synaptic responses; (2) an increase in excitatory synaptic responses; or (3) alterations to intrinsic neuronal excitability. Numerous studies have demonstrated that hearing loss results in changes to all three of these processes, suggesting that central gain enhancement may be the complex result from a confluence of synaptic and cellular changes.

Biochemical studies provide evidence for long-term alterations to inhibitory synapses at various levels of the auditory system after cochlear damage. In fact, sustained alterations in inhibitory input have been identified as peripheral as the CN (153, 154). Immunolabeling studies have demonstrated a reduction in both glycine-positive puncta and post-synaptic glycine receptor levels (155, 156), as well as a persistent decline in functional glycinergic markers, such as glycine uptake, release, and receptor binding assays (157–159). In the IC, a decrease in markers of GABAergic input has been observed as well (160–162). Interestingly, restricted cochlear damage resulted in altered GABA receptor and GAD expression in the IC limited to the region of trauma (163). Similarly, in the AC it was also shown that noise-trauma decreased inhibitory drive specifically in the region of hearing loss (164). Functional changes in inhibitory synaptic strength have been observed as well. Unilateral cochlear ablation decreased conductance and depolarized inhibitory reversal potential, thereby reducing inhibitory function in the VCN and IC (165, 166), and also disrupts GABAergic maturation in the AC (167, 168). Furthermore, direct application of salicylate to AC slices decreased evoked and mini IPSCs in pyramidal cells (169) and decreased the spiking rate of fast-spiking inhibitory interneurons in layer 2/3 while having no effect on pyramidal cell threshold (170), supporting the idea that salicylate-induced hyperactivity is mediated locally. Thus, numerous lines of evidence suggest that noise or ototoxic trauma associated with tinnitus results in decreased strength of inhibitory responses.

It is clear that the inhibitory system is disrupted by acoustic trauma and it is therefore imperative to determine how these changes impact gain enhancement. Middleton et al. used a metabolic imaging assay of neural activity in DCN slices to determine that noise-exposed mice with behavioral evidence of tinnitus had steeper input–output functions, which may be indicative of enhanced gain. They demonstrated that while blocking excitation had a similar effect on activity on control and noise-exposed mice, blocking GABAergic inhibition enhanced responses to a greater extent in control mice than in noise-damaged mice (154). These results suggest that decreased inhibition may be the predominant determinant of enhanced activity in the DCN. In another recent study, Sun (115) demonstrated that the enhancement of sound-evoked responses induced by salicylate in the AC is also likely dependent on changes to inhibition. When animals were anesthetized with isoflurane, which increases GABA-mediated inhibition, the amplitude enhancement observed in awake-animals was abolished. Further evidence for the role of GABAergic transmission in salicylate-induced enhancement comes from studies showing that local application of vigabatrin, which enhances GABA levels in the brain, suppressed the salicylate-induced enhancement of AC firing rate (138). Recent studies using optogenetic techniques have demonstrated that a subclass of inhibitory interneurons, parvalbumin positive neurons (PV+), are particularly well suited to mediate gain control (171–173). Indeed, it has been demonstrated that PV+ neurons provide dynamic gain control and shape intensity tuning in the AC (174). Furthermore, another inhibitory interneuron type, vasoactive intestinal polypeptide (VIP) expressing neurons, specialize in disinhibitory gain control by specifically inhibiting PV+ neurons (175). However, it is not known how either cell-type may be altered by hearing loss. Future studies using local, direct manipulation of inhibitory neuron subtypes in different anatomical regions is required to better understand the role of inhibition in auditory gain enhancement.

While less extensively studied, cochlear trauma has been shown to alter excitatory synaptic function as well. Alterations to glutamatergic metabolism in the auditory brainstem and midbrain have been observed as a result of cochlear trauma (176–179). Post-synaptic alterations in excitatory receptor levels, subunit composition, and cellular localization have also been documented in the CN and IC. Interestingly, while some studies observed a decrease in excitatory function (180, 181), many found that cochlear damage resulted in an increase in markers of excitatory function (182–184). Ultrastructure studies demonstrated that auditory deafferentation results in a thickening of the post-synaptic density in the VCN, suggesting an increase in post-synaptic excitatory strength (183, 185, 186). Immunogold labeling demonstrated increased α-amino-3-hydroxy-5-methyl-4-isoxazolepropionic acid (AMPA) receptor surface labeling in both the VCN and DCN days after unilateral cochlear ablation (183). Moreover, cochlear ablation increased EPSCs in the IC and AC in parallel with decreases in IPSCs (187, 188). Thus, there is concomitant bidirectionally opposed regulation of excitatory and inhibitory synaptic function in response to loss of auditory input. It is important to note that the temporal and spatial dynamics of these excitatory alterations are varied as rapid (184), transient (180, 181, 189) as well as tonotopically restricted (178) changes have been observed.

In addition to synaptic modifications, sound-evoked activity can be modulated by a neuron’s intrinsic excitability, which is largely determined by the expression or biophysical properties of voltage- and Ca^2+^-gated ion channels. Acoustic trauma has been shown to alter the intrinsic properties of granule (190) and fusiform cells (68, 191) in the DCN; these alterations were shown to occur in animals with behavioral evidence of tinnitus. Furthermore, the enhanced excitability was likely due to decreased conductance of Kv7 family of voltage-gated potassium channels (also termed KCNQ) (191). Consistent with this, channel modulators that enhance potassium currents have been shown to suppress behavioral evidence of tinnitus (192). Increased pyramidal cell excitability has also been observed in the AC specifically in the frequency region associated with hearing loss and tinnitus perception (188, 193).

### Models of central gain enhancement

The above evidence demonstrates that a multitude of synaptic and cellular changes occur after cochlear damage, which could contribute to the enhancement of sound-evoked activity observed *in vivo*. How might cochlear trauma lead to these cellular changes? Broadly speaking, there are two ways that central auditory neural activity could be altered by peripheral hearing loss. First, there are likely to be immediate reactive changes as a result of reduced auditory input. It is known that the dynamic range of central auditory neurons is rapidly modulated in response to alterations in the pattern and level of incoming sound, as well as the behavioral significance of the sound being analyzed (154, 164, 191, 194–202). As hearing loss reduces and disrupts the magnitude and features of the auditory input, central gain enhancement may be a direct consequence of an altered neuronal network displaying emergent properties because of unmasked synaptic connections. However, slower compensatory changes in response to the long-term loss of auditory input are likely to occur as well. Indeed, most sensory systems are known to exhibit long-term changes in function and connectivity in response to sensory deprivation (203–207) and auditory deprivation is known to result in alterations to neural activity in many brain areas (208). Thus, it is likely that long-term plasticity mechanisms contribute to central gain enhancement in many auditory regions as well. Here, we will review some prominent models of how central gain enhancement may arise, examine the potential mechanisms that underlie them, and determine how they relate to the cellular changes described above.

#### Divisive normalization as a canonical computation for gain control

An extraordinary feat of the auditory system is that it is able to process sounds over a wide range of amplitudes, roughly 10–12 orders of intensity (209), with high sensitivity and accuracy (210, 211). This is all the more surprising given that individual auditory neurons display a much smaller dynamic range, typically 30–50 dB. Several lines of evidence suggest that the gain of sensory neurons in the cortex can be adjusted by a computational process called divisive normalization (212). Divisive normalization is the computation of a ratio between the response of an individual neuron and a common factor, typically the summed activity of a pool of neurons. In the auditory system, there is some evidence that this type of computational strategy is at play at the level of the IC and AC, such that the input–output functions and spectro-temporal receptive fields can be modulated by background noise, which stimulates a large population pool in the auditory system (197, 200, 213–215). Such computation implies a very fast adaptation to the statistics of the sensory input. Although divisive normalization seems to be pervasive throughout sensory systems and an important basic computational strategy used in sensory gain control, it has not been fully explored in experimental model of tinnitus. Nevertheless, there is evidence that cochlear damage disrupts lateral inhibitory networks in several auditory areas, which is a key cellular mechanism underling divisive normalization models (216–218). Since lateral inhibitory networks in the auditory system extend across a wide range of frequencies, the sum of a lateral inhibitory network activated by a complex sound could conceivably represent the normalization factor.

#### Disruption of lateral inhibition after hearing loss

**Figure 7:**
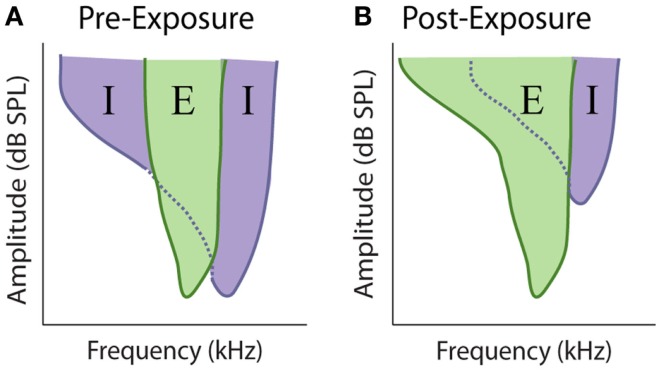
**Model of excitatory and inhibitory receptive field overlap in the auditory system**. **(A)** Under control conditions there is strong inhibition (purple) at edge of the characteristic excitatory (green) frequency. At frequencies where the threshold for inhibition is equal to or lower the threshold for excitation, the response of the neuron is inhibited, resulting in narrow excitatory tuning curves. **(B)** Noise-exposure that causes restricted cochlear damage above the excitatory characteristic frequency results in the loss of this side-band inhibition, resulting in broader excitatory tuning curves.

At every stage in the central auditory system, a given neuron’s receptive field depends on the relative strength and overlap of excitatory and inhibitory inputs (Figure 7A) (219–223). Evidence suggests that the balance between these competing excitatory and inhibitory inputs is maintained at a stable ratio (E/I ratio) (219, 221), and disruption of this balance has been proposed to underlie rapid receptive field changes seen in cortical neurons (224, 225). Therefore, the loss of peripheral inputs is likely to disrupt this E/I balance and could potentially mediate the observed alterations in auditory response properties after cochlear damage, as has been observed with denervation of somatosensory and visual systems (226–231) (Figure 7B). Indeed, one of the first proposed mechanisms for auditory gain enhancement was the loss of lateral or side-band inhibition (29, 57, 69, 59, 232).

**Figure 8:**
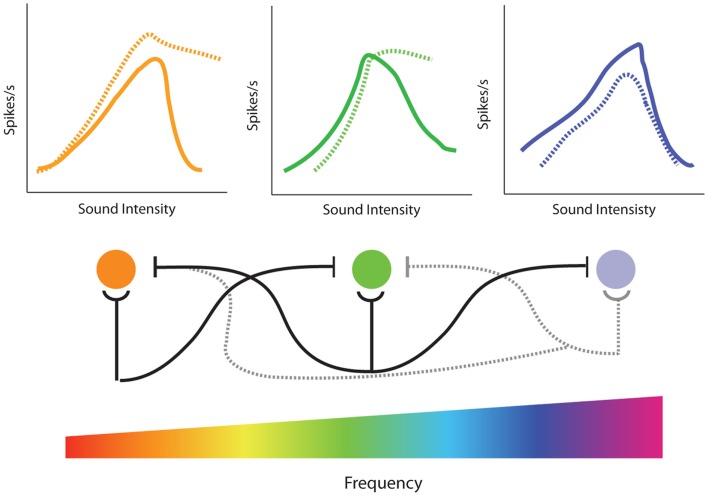
**Model of the effects of loss of lateral inhibition on rate-level functions from single-unit recordings in the inferior colliculus**. (Bottom) Tonotopic projections from the cochlea result in excitatory responses of corresponding frequency regions in the inferior colliculus (IC) (cochlear nucleus is not represented for clarity). In addition to excitatory projections (semi-circles), there are inhibitory projections (flat lines) to neighboring frequencies resulting in lateral inhibition. High-frequency hearing loss results in both the loss of excitatory projections to the corresponding tonotopic region in the IC as well as loss of inhibitory projections to surround frequencies (dotted lines). (Top) Normally, increasing sound intensity results in recruitment of lateral inhibitory projections so that many cells in the IC have non-monotonic rate-level functions (solid lines). High-frequency noise-damage not only results in decreased rate-level functions in the region of hearing loss (dotted blue line) but enhanced rate-level functions and increased monotonicity due to loss of lateral inhibition at edge frequencies (dotted orange and green lines). This loss of lateral inhibition and increase in monotonic rate-level functions could contribute to the enhancement of amplitude-level functions observed at frequencies outside of hearing loss region in the IC (see Figure 5).

There is extensive evidence demonstrating that lateral inhibition exists at many stages of the auditory system. At lower levels, such as the VCN, lateral inhibition is typically restricted to relatively narrow tonotopic regions surrounding CF (233), but at higher levels inhibitory responses are known to span large frequency regions (234). In the AC, some neurons can receive inhibitory inputs covering much of the auditory spectrum (219). Thus, restricted cochlear damage would not only result in decreased excitatory input in the region of hearing loss, but would also likely decrease inhibition at nearby, and possibly distant, frequencies (Figure 8, bottom). This loss of surround inhibition could then unmask previously inhibited excitatory responses (Figure 7B) leading to the observed gain enhancement at frequencies below the region of cochlear damage (Figure 8, top).

The model of side-band inhibition makes several predictions about auditory neuron response properties and how they will be affected by cochlear damage. First, strong lateral inhibition should result in extremely narrow tuning curves (Figure 7A), which is indeed similar to those seen in many neurons throughout the auditory system (222, 235–239). If this is the case, then loss of inhibition should result in the broadening of excitatory tuning curves (Figure 7B). In agreement with this, direct application of GABA antagonists to the AC results in dramatic expansion of receptive fields (237). The overlap of excitatory and inhibitory inputs should also have an impact on rate-intensity functions (Figure 8). Spike rates increase with sound levels over low-to-moderate intensities where excitatory inputs exceed inhibition. However, at high levels, inhibitory circuits are recruited and overwhelm excitation resulting in a suppression of firing at high intensities. Therefore, another prominent characteristic of central auditory neurons with strong lateral inhibition is a non-monotonic rate-level function (Figure 8, top), and indeed these are observed in several auditory areas (69, 71, 195, 240, 241). Thus, another prediction of the side-band inhibition model is that neurons with non-monotonic rate-level functions should be more susceptible to gain enhancement than monotonic neurons. Furthermore, since loss of lateral inhibition should result in decreased suppression at high stimulation intensities, non-monotonic units should become more monotonic with acoustic trauma to frequencies near its CF (Figure 8, dashed lines).

To test these predictions, single-unit recordings from the IC and DCN (69, 71) were performed before and immediately after a traumatizing tone (100–117 dB, 15–30 min, 1/3–1 octave above CF). In approximately 40% of IC neurons and 25% of DCN neurons, it was found that while noise-exposure far above CF had no effect on CF-threshold or high-frequency tuning curve slope, there was substantial extension of the low frequency tail of the tuning curve below-CF, particularly for neurons that initially had extremely narrow tuning curves. Furthermore, spike rate-level functions for these units were higher at all intensities, and the enhancement was greater at high stimulation intensities resulting in more monotonic-like rate-level functions. Importantly, neurons in the IC and DCN that were unaffected by the traumatizing exposure tended to already have monotonic rate-level functions and broad, open-V tuning curves, suggesting they lacked strong lateral inhibitory inputs. Carboplatin-induced IHC loss was shown to result in a similar increase in monotonicity for units in the IC, consistent with the effects of carboplatin on amplitude-level functions (242). Similar results were observed in the AC using a forward masking protocol (195). Interestingly, in this study, neurons within the region of hearing loss were affected more than those outside the region, similar to the differences in spectral profile of gain enhancement between the AC and IC (Figure 5). A recent study showed that while monotonic primary-like neurons in the VNC had reduced rate-level functions after noise-trauma, chopper-like cells that were predominantly non-monotonic showed enhancement (66). Thus, the presence or lack of strong lateral inhibition may underlie the cell-type specificity of gain enhance in this region.

Extracellular unit recordings have proved to be an extremely useful technique for inferring the presence of lateral inhibition. They are, however, still an indirect measure of a neurons E/I ratio. More recent studies have used *in vivo* whole cell recordings to directly measure tone-evoked excitatory and inhibitory synaptic inputs in auditory neurons. Consistent with extracellular recordings, many neurons in the IC display robust side-band inhibition (243). Surprisingly, in the AC it appears that excitatory and inhibitory inputs to a neuron are co-tuned, challenging classical models of lateral inhibition (219, 221). However, other studies have demonstrated this balance is only approximate and intracortical inhibition can laterally sharpen frequency tuning (222). In agreement with lateral inhibition models, acoustic trauma resulted in loss of inhibition at frequencies well below the trauma tone frequency (231). However, the same study also found a robust increase in inhibition at frequencies at the edge of the threshold shift. Furthermore, there were alterations in excitatory synaptic responses that, while smaller in magnitude, still had significant effects on response properties. These results suggest that, at least in the AC, alterations in auditory response and receptive field properties include unmasking by selective loss of inhibition, but are ultimately mediated by distinct interactions between synaptic excitation and inhibition.

#### Multiplicative rescaling and gain control

Another model for central gain enhancement advocates that the observed enhancement is largely a compensatory mechanism to preserve neural coding efficiency after a loss of sensory input, from which tinnitus arises as a side-effect (244). This multiplicative central gain model argues that central sensory neurons follow a maximum information principle, which states that neurons must make optimal use of their available responses to maintain coding efficiency (245). This means that the Shannon Entropy of neural responses should be maximal. In order to satisfy this requirement, the distribution of neural responses in central auditory neurons should be uniform across a range of responses. If a given central sensory neuron receives its inputs from auditory fibers with a Gaussian distribution of firing rates, then the input–output function of this central auditory neuron should be the integral of the Gaussian distribution. In that case, the input–output function of central auditory neurons corresponds to a sigmoid function (244). There is evidence that this coding efficiency principle is prevalent in the visual system (246), and possibly in the auditory system as well (197, 213, 247–249).

According to the multiplicative gain control model, sensory deprivation must cause a leftward shift in the Gaussian distribution of inputs that central auditory neurons receives from the AN. Since central auditory neurons integrate this distribution, the input–output function of these neurons will became steeper and maximum firing rates would become slightly larger than normal, similar to what happens to rate-level functions of VCN neurons (64, 66) and DCN neurons (67, 68) after noise-trauma. In that case, the distribution of the responses of these central neurons will be uniform, and the basic requirement for coding efficiency will be maintained. Therefore, the outcome of the multiplicative gain control model is a compensatory neural sensitivity to maintain coding efficiency. However, this would also result in an amplification of spontaneous activity coming from the spared auditory fibers, which may be interpreted as a neural noise that could potentially underlie tinnitus.

#### Homeostatic plasticity and central gain enhancement

One possible mechanism for the maintenance of multiplicative gain enhancement is homeostatic plasticity (244). Homeostatic plasticity is a regulatory mechanism that allows a neuron to increase or decrease its overall activity level in response to associative changes in synaptic strength, thereby maintaining the stability of neural networks (250). The long-term loss of auditory input is therefore likely to evoke a homeostatic response, increasing neural activity in attempts to compensate for the sensory deprivation. In essence, homeostatic plasticity is a form of cellular gain control, keeping neuronal activity within its dynamic range in the face of changing input without disrupting the balance of synaptic weights. Thus, it has been proposed that hyperactivity of central auditory neurons after noise-damage may be generated by homeostatic mechanisms (251–254). An important question to address then is: do the cellular mechanisms of homeostatic plasticity fit with the known properties of the neuronal enhancement seen after hearing loss?

There are several proposed mechanisms for the maintenance of neural homeostasis. It can be maintained by modification of neuronal intrinsic excitability (255, 256), which is a relatively simple way of regulating the overall activity of a neuron while maintaining appropriate synaptic weights. Indeed, there is evidence to suggest this homeostatic mechanism occurs in the central auditory system after hearing loss (154, 164, 191, 202). More recently, synaptic scaling has been proposed as another mechanism for homeostasis (257, 258). Synaptic scaling involves the global renormalization of synaptic strength in response to long-term changes in activity. Broadly speaking, a decrease in activity leads to a subsequent cell-wide increase in synaptic strength (“scaling up”) and, conversely, an increase in activity leads to a decrement in synaptic strength (“scaling down”) (257). In this way, synaptic scaling re-establishes a normal range of synaptic strength while maintaining the relative difference in strength between synapses.

Several studies have shown scaling-like changes occur in the IC and AC after cochlear ablation in young gerbils (167, 187, 188). However, since synaptic scaling mechanism are known to be age-dependent (257, 258), it is important to determine if similar changes are observed in adult animals. There is some evidence to suggest that the synaptic changes in adult animals after cochlear trauma described above involve synaptic scaling. A defining characteristic of synaptic scaling is that it is multiplicative in nature, i.e., all synapses will be affected in a similar manner. Indeed, a global reduction in inhibitory synaptic strength (measure electrophysiologically by miniature IPSP amplitude) is observed in the AC after acoustic trauma in adult animals, specifically in the region of hearing loss and tinnitus perception (164). In the DCN, ultrastructure studies have observed a redistribution of AMPARs in fusiform cells not only at AN synapses, where there is decreased peripheral input, but also at parallel fiber (PF) synapses, which does not receive direct auditory input (183). Functionally, acoustic damage is not only associated with increased rate-level functions in the DCN but also a conversion of PF-mediated suppression of fusiform cells to excitation (67). These studies suggest that the loss of auditory input may indeed result in neuron-wide changes in synaptic function that is indicative of homeostatic synaptic scaling. Moreover, these results are interesting in context of somatic modulation of tinnitus (259, 260), as PF input to the DCN is the first site of somatosensory and auditory stimulus convergence.

Finally, a third mechanism for homeostatic regulation involves metaplasticity; that is, previous activity dictates the level of future plasticity in a neuron (261). In this manner, the loss of auditory input may fundamentally alter the ability of neurons in the central auditory system to undergo further synaptic changes. Indeed, plasticity rules at PF synapses in fusiform cells have been shown to be qualitatively altered after hearing damage and this metaplastic change could contribute to the changes in PF function described above (262). Immunogold labeling studies demonstrate that hearing loss not only alters total levels of AMPARs in the CN, but causes a change in subunit distribution (183). Subunit reorganization can modulate channel conductance and Ca^2+^ permeability, and therefore might alter the ability of these synapses to exhibit plasticity. Similar qualitative changes in synaptic plasticity have been observed in layer 5 of the AC, however this was once again observed in juvenile animals (263). Thus, beyond examining how acoustic trauma directly alters synaptic strength, further studies should examine how the ability of future synaptic modification is altered by loss of peripheral input as well.

It is also likely that multiple homeostatic mechanisms can operate in parallel. Indeed, in the visual cortex, ocular dominance shifts in response to visual deprivation has been shown to be mediated by a cohort of changes encompassing both metaplastic and synaptic scaling-like mechanisms (264). In the AC, high-frequency hearing loss results in a global reduction in inhibitory synaptic strength as well as increased neuronal excitability within the region of acoustic trauma, suggesting both synaptic scaling and homeostatic intrinsic plasticity mechanisms are in operation (164, 193). Meanwhile, the same study saw increased pyramidal cell EPSCs and IPSCs in the low frequency region, which correlated with tonotopic map expansion, likely reflecting metaplastic changes (164). These results suggest that altered auditory input affects central auditory neuron in a variety of ways and further work is needed to fully understand the overall consequences of these changes and how they may relate to central gain enhancement.

## Central Gain, Tinnitus, and Hyperacusis

Central gain enhancement has been observed in many auditory areas in response to a variety of acoustic or ototoxic insults and a myriad of potential mechanisms have been implicated in these changes. The question remains, however, as to how these changes may contribute to tinnitus and/or hyperacusis. According to the Central Gain Model, the central auditory system recalibrates its mean firing rate activity to a new “set-point” after a lack of sensory input, thereby generating an amplification of neural noise, which would be perceived as tinnitus. Importantly, this neuronal recalibration would also result in an amplification of incoming sensory signals, which may underlie loudness intolerance and hyperacusis that also often accompanies hearing loss. Thus, an attractive aspect of the Central Gain model is that it could account for both tinnitus and hyperacusis.

### Central gain enhancement and hyperacusis

Loudness perception is dynamic. Modulating the background noise levels during the presentation of a sound can change the perceived loudness of that sound (197, 244, 265). This is thought to be accomplished by gain modulation in the auditory system. Indeed, central auditory neurons can adapt their sensitivity to auditory input based on the sound level statistics, allowing them to maintain a relatively stable range of activity thereby preserving neural coding efficiency (200). Consistent with this model, psychoacoustic studies have determined that loudness perception is more closely correlated with the level of sound-evoked activity in the CNS than with the absolute sound level (266, 267). Thus, central gain modulation is likely to be intimately linked to loudness perception, suggesting that central gain enhancement may manifest as hypersensitivity to loudness, i.e., hyperacusis. Indeed, both human and animal studies have found that enhanced sound-evoked activity is correlated with hyperacusis-like behavior (7, 32, 35). One recent imaging study found that people with hyperacusis, regardless of the presence of tinnitus, had greater sound-evoked neural activity in the auditory midbrain, thalamus, and primary AC than normal patients matched for hearing loss, demonstrating a relationship between enhanced central gain and hyperacusis (7). Interestingly, the presence of tinnitus in the same population was only correlated with increased sound-evoked activity in the AC but not lower auditory structures. These results suggest that central gain enhancement in distinct auditory structures may differentially contribute to tinnitus and hyperacusis.

### Central gain enhancement and tinnitus

The multiplicative gain control model described above suggests that auditory deprivation results in an increase in neuronal sensitivity in attempts to compensate for lost inputs. It was proposed that tinnitus is a side-effect of this hyperactivity due to the amplification of spontaneous firing coming from spared auditory fibers (244). A critical aspect of this model is the role of “neural noise” in tinnitus generation. The central auditory system is constantly receiving input even in silence. Spontaneous firing rates from the AN average around 50 spikes/s and can be as high as 120 spikes/s in a sound isolation booth (244). Thus, there is a baseline level of activity (i.e., neural noise) that defines “silence.” Central gain enhancement not only results in increased sensitivity to sound-evoked activity, but purportedly amplifies spontaneous activity as well, leading to auditory perception of this neural noise. Indeed, hearing loss has been shown to result in increased spontaneous activity in the central auditory system, particularly in the DCN, despite decreased spontaneous firing in the AN (24, 25). While this model proposes that neural noise may originate in the periphery, this must be reconciled with the fact that AN resection, which abolishes spontaneous activity, does not consistently abolish tinnitus (16).

Alternatively, increased neural noise can be generated centrally (e.g., in the DCN or IC) in response to gain enhancement, in order to maintain the proper ratio between evoked and spontaneous activity (i.e., signal to noise ratio) (268). It is important to bear in mind that while enhanced sound-evoked responses are observed relatively quickly (Figure 4), spontaneous hyperactivity observed in DCN within the region of hearing loss takes several days post-noise-exposure to arise. Thus, it is unlikely that this increase in neural noise can account for acute tinnitus, which arises immediately after noise-exposure; however, that does not preclude involvement with slower developing chronic tinnitus. More studies are needed to determine the biological origins of neural noise in order to explicitly test these theoretical models and determine the mechanistic relationship between central gain and tinnitus. Nevertheless, behavioral studies have suggested a correlation between central gain enhancement and tinnitus.

Human brain imaging studies with PET provided some of the earliest support for the Central Gain model of hyperacusis and tinnitus, finding greater sound-evoked activity in the AC of tinnitus patients compared to normal controls (269). Similar results have been observed in more recent imaging studies, with tinnitus patients exhibiting enhanced activity in several central auditory structures, including the IC, MGB, and AC (7, 270, 271). ABR recordings from tinnitus patients have also lent support to the notion that central gain enhancement is associated with tinnitus. These studies have demonstrated that despite having decreased wave I amplitude, which is indicative of decreased AN output, tinnitus patients have normal or even enhanced responses for later waves that reflect activity from more central structures, such as the brainstem and midbrain (253, 254, 272, 273). Interestingly, while both tinnitus and hearing loss-matched non-tinnitus subjects had increased thresholds and reduced wave I amplitude compared to a third, normal hearing control group, only tinnitus subjects exhibited enhanced wave V activity suggesting that central gain enhancement is not merely a reflection of hearing loss but specifically related to the presence of tinnitus (274).

Several animal studies have demonstrated a correlation between gain enhancement and tinnitus-like behavior as well. High doses of salicylate not only results in gain enhancement in the AC, MGB, amygdala, and IC, but also behavioral evidence of tinnitus and hyperacusis (35, 109, 192). A similar correlation between enhanced responses in the AC and tinnitus-like behavior has been observed with noise-damage (275); pairing tones with vagal nerve stimulation effectively reversed both behavioral evidence of tinnitus and several neurophysiological changes associated with the noise-damage, including hyperactivity of sound-evoked responses. It should be noted however, that the frequency of sound-evoked hyperactivity was only weakly correlated with tinnitus pitch in this study. Enhanced rate-level functions in the DCN are also correlated with tinnitus behavior (67, 68), and there is some evidence that neuronal enhancement was restricted to the region of hearing loss and tinnitus pitch (68). Further studies are needed to correlate neuronal enhancement to the pitch of tinnitus.

While these human and animal studies demonstrate a correlation between tinnitus and central gain enhancement, there are some caveats that must be considered. First, tinnitus rarely occurs in isolation, as it is consistently associated with peripheral hearing loss and is also often associated with hyperacusis (2, 6, 7, 268, 276, 277). In fact, these two disorders are highly correlated, with an estimated 40% of patients experiencing tinnitus also suffering from hyperacusis (278) and, conversely, 87% of the patients experiencing hyperacusis also being diagnosed with tinnitus. While many studies examining the correlation between tinnitus and central gain enhancement have controlled for hearing loss, relatively few have attempted to account for the potential contribution of hyperacusis. Thus, it is unclear if central gain enhancement is more correlated with tinnitus, hyperacusis, or both. This is compounded by the fact that many patients are unaware that they have hyperacusis or loudness tolerance problems unless explicitly tested (7, 279). Indeed, a recent study has demonstrated that hyperacusis was more common in individuals with tinnitus compared with normal hearing individuals, even when hearing loss was controlled for (280).

A second limitation to the correlational studies described above is that central gain enhancement is likely to occur with several other neurophysiological alterations associated with hearing loss, such as increased spontaneous firing rates, enhanced neural synchrony, and tonotopic map reorganization. The simultaneous occurrence of several neurophysiological changes makes it difficult to determine which changes are causally related to tinnitus and/or hyperacusis. Thus, it is important to examine the relationship between central gain enhancement and other neurophysiological changes with hearing loss; are they different consequences of the same underlying physiological changes or distinct processes that occur in parallel, and if so, how do they interact with each other? As addressed above, there is likely an intimate relationship between central gain enhancement and increased spontaneous activity, however, the exact nature of this relationship remains to be determined. Enhanced neural synchrony is another common alteration observed with hearing loss. In particular, there are several reports that tinnitus patients exhibit increased EEG oscillatory activity, especially in the gamma frequency range, which is indicative of increased synchrony of neuronal assemblies (10, 23, 281). It has been proposed that gamma oscillations may facilitate the transmission of information across neuronal areas by enabling large neuronal networks to fire in a specific phase of the gamma frequency cycle (282–285). As such, it is possible that gamma oscillations could entrain specific neuronal networks across the tonotopic map and underlie the neuronal enhancement observed in these areas. However, to our knowledge, no studies have attempted to directly examine the correlation between increased gamma frequency band power and neuronal gain enhancement in tinnitus patients. It is also conceivable that the enhancement of sound-evoked responses at frequencies outside the region of hearing loss is permissive to tonotopic reorganization, however, once again there is little work directly examining the relationship between these processes. Such lines of research are important to unveil the relationship between the numerous neurophysiological changes observed with hearing loss.

## Conclusion

The results reviewed here suggest that gain enhancement occurs at many different levels of the central auditory system, and even regions outside the classical auditory pathway, in response to cochlear damage. However, the temporal dynamics and spectral profile of these changes vary between auditory structures and types of trauma. These differences suggest that gain enhancement is not passively transmitted from lower to higher levels of the ascending auditory system, but is likely to occur at multiple levels concurrently and is potentially modified by descending feedback projections as well. Simultaneous, longitudinal chronic electrophysiological recordings from multiple areas, ideally spanning tonotopic regions, coupled with fine-tuned hearing assessment will allow for better understanding of the temporal and spectral aspects of central gain enhancement and their relationship to tinnitus and hyperacusis. The variations in spectral and temporal dynamics of central gain enhancement further suggest that there are likely multiple neuronal mechanisms involved in its expression. For instance, the rapid unmasking of established inputs via loss of lateral inhibition may account for the immediate neuronal enhancement observed at frequencies outside the region of hearing loss, while homeostatic plasticity may mediate the slower gain changes within hearing loss regions seen with more intense noise-damage. It is tempting to speculate that differences in the spectral and temporal aspects of central gain enhancement may correlate with the differences in the onset and pitch of tinnitus. Understanding how gain changes are implemented at the cellular-level will allow for the development of advanced pharmacological and genetic tools necessary to elucidate the role of gain enhancement in tinnitus and hyperacusis and, most importantly, lead to novel treatments for these disorders.

## Author Contributions

Benjamin D. Auerbach, Paulo Vianney Rodrigues, and Richard J. Salvi wrote the manuscript and approved the final version.

## Conflict of Interest Statement

The authors declare that the research was conducted in the absence of any commercial or financial relationships that could be construed as a potential conflict of interest.

## References

[1] Rybak LP Ramkumar V. Ototoxicity. Kidney Int (2007) 72:931–5. 10.1038/sj.ki.5002434 17653135

[2] Baguley DM. Hyperacusis. J R Soc Med (2003) 96:582–5. 10.1258/jrsm.96.12.582 14645606 PMC539655

[3] Hoffman HJ Reed GW. Epidemiology of tinnitus. In: Snow J, editor. Tinnitus Theory and Management. Hamilton, ON: BC Decker (2004). p. 16–41

[4] Baguley D McFerran D Hall D. Tinnitus. Lancet (2013) 382:1600–7. 10.1016/S0140-6736(13)60142-7 23827090

[5] Shargorodsky J Curhan GC Farwell WR. Prevalence and characteristics of tinnitus among US adults. Am J Med (2010) 123:711–8. 10.1016/j.amjmed.2010.02.015 20670725

[6] Andersson G Lindvall N Hursti T Carlbring P. Hypersensitivity to sound (hyperacusis): a prevalence study conducted via the Internet and post. Int J Audiol (2002) 41:545–54. 10.3109/14992020209056075 12477175

[7] Gu JW Halpin CF Nam E-C Levine RA Melcher JR. Tinnitus, diminished sound-level tolerance, and elevated auditory activity in humans with clinically normal hearing sensitivity. J Neurophysiol (2010) 104(6):3361–70. 10.1152/jn.00226.2010 20881196 PMC3007631

[8] Fausti SA Wilmington DJ Gallun FJ Myers PJ Henry JA. Auditory and vestibular dysfunction associated with blast-related traumatic brain injury. J Rehabil Res Dev (2009) 46:797–810. 10.1682/JRRD.2008.09.0118 20104403

[9] Eggermont JJ Roberts LE. The neuroscience of tinnitus. Trends Neurosci (2004) 27:676–82. 10.1016/j.tins.2004.08.010 15474168

[10] Weisz N Hartmann T Dohrmann K Schlee W Norena A. High-frequency tinnitus without hearing loss does not mean absence of deafferentation. Hear Res (2006) 222:108–14. 10.1016/j.heares.2006.09.003 17079102

[11] Kiang NY Moxon EC Levine RA. Auditory-nerve activity in cats with normal and abnormal cochleas. *Sensorineural Hearing Loss*. Ciba Foundation Symposium (1970). p. 241–73 10.1002/9780470719756.ch15 5210916

[12] Lepage EL. Frequency-dependent self-induced bias of the basilar-membrane and its potential for controlling sensitivity and tuning in the mammalian cochlea. J Acoust Soc Am (1987) 82:139–54. 10.1121/1.395557 3624635

[13] Lepage EL. Functional-role of the olivocochlear bundle: a motor unit in the mammalian cochlea. Hear Res (1989) 38:177–98. 10.1016/0378-5955(89)90064-6 2708162

[14] Jastreboff PJ. Phantom auditory perception (tinnitus): mechanisms of generation and perception. Neurosci Res (1990) 8:221–54. 10.1016/0168-0102(90)90031-9 2175858

[15] Dallos P Harris D. Properties of auditory nerve responses in absence of outer hair cells. J Neurophysiol (1978) 41(2):365–83. 650272 10.1152/jn.1978.41.2.365

[16] House JW Brackmann DE. Tinnitus: surgical treatment. Ciba Found Symp (1981) 85:204–16. 6915835 10.1002/9780470720677.ch12

[17] Feldmann H. Homolateral and contralateral masking of tinnitus by noise-bands and by pure tones. Audiology (1971) 10:138–44. 10.3109/00206097109072551 5163656

[18] Flor H Elbert T Muhlnickel W Pantev C Wienbruch C Taub E. Cortical reorganization and phantom phenomena in congenital and traumatic upper-extremity amputees. Exp Brain Res (1998) 119:205–12. 10.1007/s002210050334 9535570

[19] Muhlnickel W Elbert T Taub E Flor H. Reorganization of auditory cortex in tinnitus. Proc Natl Acad Sci U S A (1998) 95:10340–3. 10.1073/pnas.95.17.10340 9707649 PMC21510

[20] Rauschecker JP. Auditory cortical plasticity: a comparison with other sensory systems. Trends Neurosci (1999) 22:74–80. 10.1016/S0166-2236(98)01303-4 10092047

[21] Noreña AJ Eggermont JJ. Changes in spontaneous neural activity immediately after an acoustic trauma: implications for neural correlates of tinnitus. Hear Res (2003) 183:137–53. 10.1016/S0378-5955(03)00225-9 13679145

[22] Llinás R Urbano FJ Leznik E Ramírez RR van Marle HJ. Rhythmic and dysrhythmic thalamocortical dynamics: GABA systems and the edge effect. Trends Neurosci (2005) 28:325–33. 10.1016/j.tins.2005.04.006 15927689

[23] Weisz N Müller S Schlee W Dohrmann K Hartmann T Elbert T. The neural code of auditory phantom perception. J Neurosci (2007) 27:1479–84. 10.1523/JNEUROSCI.3711-06.2007 17287523 PMC6673575

[24] Kaltenbach JA Afman CE. Hyperactivity in the dorsal cochlear nucleus after intense sound exposure and its resemblance to tone-evoked activity: a physiological model for tinnitus. Hear Res (2000) 140:165–72. 10.1016/S0378-5955(99)00197-5 10675644

[25] Mulders WH Robertson D. Hyperactivity in the auditory midbrain after acoustic trauma: dependence on cochlear activity. Neuroscience (2009) 164:733–46. 10.1016/j.neuroscience.2009.08.036 19699277

[26] Rauschecker JP Leaver AM Mühlau M. Tuning out the noise: limbic-auditory interactions in tinnitus. Neuron (2010) 66:819–26. 10.1016/j.neuron.2010.04.032 20620868 PMC2904345

[27] De Ridder D Elgoyhen AB Romo R Langguth B. Phantom percepts: tinnitus and pain as persisting aversive memory networks. Proc Natl Acad Sci U S A (2011) 108:8075–80. 10.1073/pnas.1018466108 21502503 PMC3100980

[28] Henry JA Roberts LE Caspary DM Theodoroff SM Salvi RJ. Underlying mechanisms of tinnitus: review and clinical implications. J Am Acad Audiol (2014) 25:5–22. 10.3766/jaaa.25.1.2 24622858 PMC5063499

[29] Salvi R Henderson D Boettcher F Powers N. Functional changes in central auditory pathways resulting from cochlear diseases. In: Katz J Stecker N Henderson D, editors. Central Auditory Processing: A Transdisciplinary View. St. Louis, MO: Mosby Year Book, Inc (1992). p. 47–60

[30] Sun W Zhang L Lu J Yang G Laundrie E Salvi R. Noise exposure-induced enhancement of auditory cortex response and changes in gene expression. Neuroscience (2008) 156:374–80. 10.1016/j.neuroscience.2008.07.040 18713646 PMC2573047

[31] Chen G-D Manohar S Salvi R. Amygdala hyperactivity and tonotopic shift after salicylate exposure. Brain Res (2012) 1485:63–76. 10.1016/j.brainres.2012.03.016 22464181 PMC5319430

[32] Sun W Deng A Jayaram A Gibson B. Noise exposure enhances auditory cortex responses related to hyperacusis behavior. Brain Res (2012) 1485:108–16. 10.1016/j.brainres.2012.02.008 22402030

[33] Chen G-D Stolzberg D Lobarinas E Sun W Ding D Salvi R. Salicylate-induced cochlear impairments, cortical hyperactivity and re-tuning, and tinnitus. Hear Res (2013) 295:100–13. 10.1016/j.heares.2012.11.016 23201030 PMC4191647

[34] Niu Y Kumaraguru A Wang R Sun W. Hyperexcitability of inferior colliculus neurons caused by acute noise exposure. J Neurosci Res (2013) 91:292–9. 10.1002/jnr.23152 23151900

[35] Chen G-D Radziwon KE Kashanian N Manohar S Salvi R. Salicylate-induced auditory perceptual disorders and plastic changes in nonclassical auditory centers in rats. Neural Plast (2014) 2014:18. 10.1155/2014/658741 24891959 PMC4033555

[36] Liberman MC. Efferent synapses in the inner hair cell area of the cat cochlea: an electron microscopic study of serial sections. Hear Res (1980) 3:189–204. 10.1016/0378-5955(80)90007-6 7440423

[37] Liberman MC. The cochlear frequency map for the cat-labeling auditory nerve fibers of known characteristic frequency. J Acoust Soc Am (1982) 72:1441–9. 10.1121/1.388677 7175031

[38] Liberman MC. Single-neuron labeling in the cat auditory nerve. Science (1982) 216:1239–41. 10.1126/science.7079757 7079757

[39] Glowatzki E Fuchs PA. Transmitter release at the hair cell ribbon synapse. Nat Neurosci (2002) 5:147–54. 10.1038/nn796 11802170

[40] Polley DB Read HL Storace DA Merzenich MM. Multiparametric auditory receptive field organization across five cortical fields in the albino rat. J Neurophysiol (2007) 97:3621–38. 10.1152/jn.01298.2006 17376842

[41] Romanski LM Averbeck BB. The primate cortical auditory system and neural representation of conspecific vocalizations. Annu Rev Neurosci (2009) 32:315–46. 10.1146/annurev.neuro.051508.135431 19400713 PMC2767298

[42] Medalla M Barbas H. Specialized prefrontal “auditory fields”: organization of primate prefrontal-temporal pathways. Front Neurosci (2014) 8:77. 10.3389/fnins.2014.00077 24795553 PMC3997038

[43] Ledoux JE Farb C Ruggiero DA. Topographic organization of neurons in the acoustic thalamus that project to the amygdala. J Neurosci (1990) 10:1043–54. 2158523 10.1523/JNEUROSCI.10-04-01043.1990 PMC6570207

[44] LeDoux JE Farb CR Romanski LM. Overlapping projections to the amygdala and striatum from auditory processing areas of the thalamus and cortex. Neurosci Lett (1991) 134:139–44. 10.1016/0304-3940(91)90526-Y 1815147

[45] Budinger E Heil P Hess A Scheich H. Multisensory processing via early cortical stages: connections of the primary auditory cortical field with other sensory systems. Neuroscience (2006) 143:1065–83. 10.1016/j.neuroscience.2006.08.035 17027173

[46] Leaver AM Renier L Chevillet MA Morgan S Kim HJ Rauschecker JP. Dysregulation of limbic and auditory networks in tinnitus. Neuron (2011) 69:33–43. 10.1016/j.neuron.2010.12.002 21220097 PMC3092532

[47] De Ridder D Vanneste S Weisz N Londero A Schlee W Elgoyhen AB An integrative model of auditory phantom perception: tinnitus as a unified percept of interacting separable subnetworks. Neurosci Biobehav Rev (2014) 44:16–32. 10.1016/j.neubiorev.2013.03.021 23597755

[48] Salvi RJ Hamernik RP Henderson D. Auditory nerve activity and cochlear morphology after noise exposure. Arch Otorhinolaryngol (1979) 224:111–6. 10.1007/BF00455233 485937

[49] Lonsbury-Martin BL Martin GK. Effects of moderately intense sound on auditory sensitivity in rhesus monkeys: behavioral and neural observations. J Neurophysiol (1981) 46(3):563–86 7299434 10.1152/jn.1981.46.3.563

[50] Saunders JC James R Bock GR Chen CS. Effects of priming for audiogenic seizure on auditory evoked-responses in cochlear nucleus and inferior colliculus of Balc/c mice. Exp Neurol (1972) 37:388–94. 10.1016/0014-4886(72)90082-9 4637958

[51] Henry KR Saleh M. Recruitment deafness: functional effect of priming-induced audiogenic seizures in mice. J Comp Physiol Psychol (1973) 84:430–5. 10.1037/h0035264) 4353282

[52] Gerken GM Saunders SS Paul RE. Hypersensibility to electrical-stimulation of auditory nuclei follows hearing-loss in cats. Hear Res (1984) 13:249–59. 10.1016/0378-5955(84)90078-9 6735932

[53] Gerken GM Saunders SS Simhadrisumithra R Bhat KH. Behavioral thresholds for electrical-stimulation applied to auditory brain-stem nuclei in cat are altered by injurious and noninjurious sound. Hear Res (1985) 20:221–31. 10.1016/0378-5955(85)90027-9 4086384

[54] Popelar J Hartmann R Syka J Klinke R. Middle latency responses to acoustical and electrical stimulation of the cochlea in cats. Hear Res (1995) 92:63–77. 10.1016/0378-5955(95)00199-9 8647747

[55] Popelar J Syka J Berndt H. Effect of noise on auditory evoked responses in awake guinea pigs. Hear Res (1987) 26:239–47. 10.1016/0378-5955(87)90060-8 3583925

[56] Salvi RJ Saunders SS Gratton MA Arehole S Powers N. Enhanced evoked response amplitudes in the inferior colliculus of the chinchilla following acoustic trauma. Hear Res (1990) 50:245–57. 10.1016/0378-5955(90)90049-U 2076976

[57] Salvi R Powers N Saunders S Boettcher F Clock A. Enhancement of evoked response amplitude and single unit activity after noise exposure. In: Dancer A Henderson D Salvi RJ Hamernik R, editors. Noise-Induced Hearing Loss. St. Louis, MO: Mosby-Year Book, Inc (1992). p. 156–71

[58] Salvi RJ Lockwood AH Burkard RF. Neural plasticity and tinnitus. In: Tyler R, editor. Tinnitus Handbook. San Diego, CA: Singular Publishing Group, Inc (2000). p. 123–48

[59] Salvi RJ Wang J Ding D. Auditory plasticity and hyperactivity following cochlear damage. Hear Res (2000) 147:261–74. 10.1016/S0378-5955(00)00136-2 10962190

[60] Wang J Ding D Salvi RJ. Functional reorganization in chinchilla inferior colliculus associated with chronic and acute cochlear damage. Hear Res (2002) 168:238–49. 10.1016/S0378-5955(02)00360-X 12117524

[61] Mitzdorf U. Current source-density method and application in cat cerebral cortex: investigation of evoked potentials and EEG phenomena. Physiol Rev (1985) 65(1):37–100 3880898 10.1152/physrev.1985.65.1.37

[62] Mitzdorf U. Properties of the evoked potential generator: current source density analysis of evoked-potentials in the cat cortex. Int J Neurosci (1987) 33:33–59. 10.3109/00207458708985928 3610492

[63] Logothetis NK. The neural basis of the blood-oxygen-level-dependent functional magnetic resonance imaging signal. Philos Trans R Soc Lond B Biol Sci (2002) 357:1003–37. 10.1098/rstb.2002.1114 12217171 PMC1693017

[64] Boettcher FA Salvi RJ. Functional changes in the ventral cochlear nucleus following acute acoustic overstimulation. J Acoust Soc Am (1993) 94:2123–34. 10.1121/1.407484 8227752

[65] Helfert RH Snead CR Altschuler RA. The ascending auditory pathways. In: Altschuker RA Bobbin RP Clopton BM Hoffman D, editors. Neurobiology of Hearing Series: Neurobiology of Hearing: The Central Auditory System. New York, NY: Raven Press (1991). p. 1–26

[66] Cai S Ma W-L Young E. Encoding intensity in ventral cochlear nucleus following acoustic trauma: implications for loudness recruitment. J Assoc Res Otolaryngol (2009) 10:5–22. 10.1007/s10162-008-0142-y 18855070 PMC2644394

[67] Dehmel S Pradhan S Koehler S Bledsoe S Shore S. Noise overexposure alters long-term somatosensory-auditory processing in the dorsal cochlear nucleus-possible basis for tinnitus-related hyperactivity? J Neurosci (2012) 32:1660–71. 10.1523/JNEUROSCI.4608-11.2012 22302808 PMC3567464

[68] Brozoski TJ Bauer CA Caspary DM. Elevated fusiform cell activity in the dorsal cochlear nucleus of chinchillas with psychophysical evidence of tinnitus. J Neurosci (2002) 22:2383–90. 11896177 10.1523/JNEUROSCI.22-06-02383.2002 PMC6758251

[69] Salvi RJ Wang J Powers NL. Plasticity and reorganization in the auditory brainstem: implications for tinnitus. In: Reich G Vernon J, editors. Proceedings of the Fifth International Tinnitus Seminar, Portland, Oregon, June 12-15, 1995. Portland, OR: American Tinnitus Association (1996). p. 457–66

[70] Salvi RJ Wang J Powers N. Rapid functional reorganization in the inferior colliculus and cochlear nucleus after acute cochlear damage. In: Salvi RJ Henderson D Fiorino F Colletti V, editors. Auditory System Plasticity and Regeneration. New York, NY: Thieme Medical Publishers (1996). p. 275–96

[71] Wang J Salvi RJ Powers N. Plasticity of response properties of inferior colliculus neurons following acute cochlear damage. J Neurophysiol (1996) 75:171–83. 8822550 10.1152/jn.1996.75.1.171

[72] Syka J Rybalko N Popelar J. Enhancement of the auditory-cortex evoked – responses is awake guinea-pigs after noise exposure. Hear Res (1994) 78:158–68. 10.1016/0378-5955(94)90021-3 7982808

[73] Syka J Rybalko N. Threshold shifts and enhancement of cortical evoked responses after noise exposure in rats. Hear Res (2000) 139:59–68. 10.1016/S0378-5955(99)00175-6 10601713

[74] Noreña AJ Moffat G Blanc JL Pezard L Cazals Y. Neural changes in the auditory cortex of awake guinea pigs after two tinnitus inducers: salicylate and acoustic trauma. Neuroscience (2010) 166:1194–209. 10.1016/j.neuroscience.2009.12.063 20096752

[75] Popelár J Nwabueze-Ogbo FC Syka J. Changes in neuronal activity of the inferior colliculus in rat after temporal inactivation of the auditory cortex. Physiol Res (2003) 52:615–28. 14535838

[76] Winer JA Larue DT. Evolution of GABAergic circuitry in the mammalian medial geniculate body. Proc Natl Acad Sci U S A (1996) 93:3083–7. 10.1073/pnas.93.7.3083 8610172 PMC39765

[77] Winer JA Saint Marie RL Larue DT Oliver DL. GABAergic feedforward projections from the inferior colliculus to the medial geniculate body. Proc Natl Acad Sci U S A (1996) 93:8005–10. 10.1073/pnas.93.15.8005 8755593 PMC38865

[78] Peruzzi D Bartlett E Smith PH Oliver DL. A monosynaptic GABAergic input from the inferior colliculus to the medial geniculate body in rat. J Neurosci (1997) 17:3766–77. 9133396 10.1523/JNEUROSCI.17-10-03766.1997 PMC6573711

[79] Bartlett EL Smith PH. Anatomic, intrinsic, and synaptic properties of dorsal and ventral division neurons in rat medial geniculate body. J Neurophysiol (1999) 81(5):1999–2016. 10322042 10.1152/jn.1999.81.5.1999

[80] Suga N Gao E Zhang Y Ma X Olsen JF. The corticofugal system for hearing: recent progress. Proc Natl Acad Sci U S A (2000) 97:11807–14. 10.1073/pnas.97.22.11807 11050213 PMC34353

[81] Zhang Y Suga N. Modulation of responses and frequency tuning of thalamic and collicular neurons by cortical activation in mustached bats. J Neurophysiol. (2000) 84(1):325–33 10899207 10.1152/jn.2000.84.1.325

[82] Liu X Yan Y Wang Y Yan J. Corticofugal modulation of initial neural processing of sound information from the ipsilateral ear in the mouse. PLoS One (2010) 5:e14038. 10.1371/journal.pone.0014038 21124980 PMC2987806

[83] Tang J Yang W Suga N. Modulation of thalamic auditory neurons by the primary auditory cortex. J Neurophysiol (2012) 108:935–42. 10.1152/jn.00251.2012 22552191 PMC3424095

[84] Popelar J Grecova J Rybalko N Syka J. Comparison of noise-induced changes of auditory brainstem and middle latency response amplitudes in rats. Hear Res (2008) 245:82–91. 10.1016/j.heares.2008.09.002 18812219

[85] Roberts LE Eggermont JJ Caspary DM Shore SE Melcher JR Kaltenbach JA. Ringing ears: the neuroscience of tinnitus. J Neurosci (2010) 30:14972–9. 10.1523/JNEUROSCI.4028-10.2010 21068300 PMC3073522

[86] Eggermont JJ Roberts LE. The neuroscience of tinnitus: understanding abnormal and normal auditory perception. Front Syst Neurosci (2012) 6:53. 10.3389/fnsys.2012.00053 22798948 PMC3394370

[87] Liberman MC Dodds LW. Single-neuron labeling and chronic cochlear patology 2. Stereocilia damage and alterations of spontaneous discharges rates. Hear Res (1984) 16:43–53. 10.1016/0378-5955(84)90024-8 6511672

[88] Liberman MC Dodds LW. Single-neuron labeling and chronic cochlear patology 3. Stereocilia damage and alterations of threshold tuning curves. Hear Res (1984) 16:55–74. 10.1016/0378-5955(84)90024-8 6511673

[89] Liberman MC Kiang NY. Single-neuron labeling and chronic cochlear patology 4. Stereocilia damage and alterations in rate-level and phase-level functions. Hear Res (1984) 16:75–90. 10.1016/0378-5955(84)90024-8 6511674

[90] Stypulkowski PH. Mechanisms of salicylate ototoxicity. Hear Res (1990) 46:113–45. 10.1016/0378-5955(90)90144-E 2380120

[91] Wake M Takeno S Ibrahim D Harrison R Mount R. Carboplatin ototoxicity in animal model. J Laryngol Otol (1993) 107:585–9. 10.1017/S0022215100123771 15125271

[92] Takeno S Harrison RV Mount RJ Wake M Harada Y. Induction of selective inner hair cell damage by carboplatin. Scanning Microsc (1994) 8:97–106. 7973504

[93] Trautwein P Hofstetter P Wang J Salvi R Nostrant A. Selective inner hair cell loss does not alter distortion product otoacoustic emissions. Hear Res (1996) 96:71–82. 10.1016/0378-5955(96)00040-8 8817308

[94] Wang J Powers NL Hofstetter P Trautwein P Ding D Salvi R. Effects of selective inner hair cell loss on auditory nerve fiber threshold, tuning and spontaneous and driven discharge rate. Hear Res (1997) 107:67–82. 10.1016/S0378-5955(97)00020-8 9165348

[95] Wang J Ding D Salvi RJ. Carboplatin-induced early cochlear lesion in chinchillas. Hear Res (2003) 181:65–72. 10.1016/S0378-5955(03)00176-X 12855364

[96] Hofstetter P Ding D Powers N Salvi RJ. Quantitative relationship of carboplatin dose to magnitude of inner and outer hair cell loss and the reduction in distortion product otoacoustic emission amplitude in chinchillas. Hear Res (1997) 112:199–215. 10.1016/S0378-5955(97)00123-8 9367242

[97] Hofstetter P Ding D Salvi R. Magnitude and pattern of inner and outer hair cell loss in chinchilla as a function of carboplatin dose. Audiology (1997) 36:301–11. 10.3109/00206099709071981 9406619

[98] Ding DL Wang J Salvi R Henderson D Hu BH McFadden SL Selective loss of inner hair cells and type-I ganglion neurons in carboplatin-treated chinchillas. Mechanisms of damage and protection. Ann N Y Acad Sci (1999) 884:152–70. 10.1111/j.1749-6632.1999.tb08640.x 10842592

[99] Salvi RJ Ding D Wang J McFadden SL Sun W. Functional changes in peripheral and central auditory pathways flowing selective inner hair cell loss. In: Simmons DD Palmer C, editors. Seminar in Hearing; New Frontiers in the Amelioration of Hearing Loss: Part – Hair Cell Development, Regeneration, Protection, and Rescue. (Vol. 24), New York, NY: Thieme (2003). p. 135–44

[100] Hofstetter P Ding D Salvi R. Induction of spontaneous otoacoustic emissions in chinchillas from carboplatin-induced inner hair cell loss. Hear Res (2000) 150:132–6. 10.1016/S0378-5955(00)00201-X 11077198

[101] Lobarinas E Salvi R Ding D. Insensitivity of the audiogram to carboplatin induced inner hair cell loss in chinchillas. Hear Res (2013) 302:113–20. 10.1016/j.heares.2013.03.012 23566980 PMC3695223

[102] Qiu C Salvi R Ding D Burkard R. Inner hair cell loss leads to enhanced response amplitudes in auditory cortex of unanesthetized chinchillas: evidence for increased system gain. Hear Res (2000) 139:153–71. 10.1016/S0378-5955(99)00171-9 10601720

[103] Jastreboff PJ Brennan JF Coleman JK Sasaki CT. Phantom auditory sensation in rats: an animal model for tinnitus. Behav Neurosci (1988) 102:811–22. 10.1037/0735-7044.102.6.811 3214530

[104] Day RO Graham GG Bieri D Brown M Cairns D Harris G Concentration-response relationships for salicylate-induced ototoxicity in normal volunteers. Br J Clin Pharmacol (1989) 28:695–702. 10.1111/j.1365-2125.1989.tb03562.x 2611090 PMC1380040

[105] Brien JA. Ototoxicity associated with salicylate – a brief review. Drug Safety (1993) 9:143–8. 10.2165/00002018-199309020-00006 8397891

[106] Bauer CA Brozoski TJ Rojas R Boley J Wyder M. Behavioral model of chronic tinnitus in rats. Otolaryngol Head Neck Surg (1999) 121:457–62. 10.1016/S0194-5998(99)70237-8 10504604

[107] Cazals Y. Auditory sensori-neural alterations induced by salicylate. Prog Neurobiol (2000) 62:583–631. 10.1016/S0301-0082(00)00027-7 10880852

[108] Guitton MJ Caston J Ruel J Johnson RM Pujol R Puel JL. Salicylate induces tinnitus through activation of cochlear NMDA receptors. J Neurosci (2003) 23:3944–52. 12736364 10.1523/JNEUROSCI.23-09-03944.2003 PMC6742173

[109] Lobarinas E Sun W Cushing R Salvi RJ. A novel behavioral paradigm for assessing tinnitus using schedule-induced polydipsia avoidance conditioning (SIP-AC). Hear Res (2004) 190:109–14. 10.1016/S0378-5955(04)00019-X 15051133

[110] Yang G Lobarinas E Zhang LY Turner J Stolzberg D Salvi R Salicylate induced tinnitus: behavioral measures and neural activity in auditory cortex of awake rats. Hear Res (2007) 226:244–53. 10.1016/j.heares.2006.06.013 16904853

[111] Lobarinas E Sun W Stolzberg D Lu J Salvi R. Human brain imaging and animal models of tinnitus. Sem Hear (2008) 29:333–49. 10.1055/s-0028-1095893 PMC2613289 19122834

[112] Stolzberg D Salvi RJ Allman BL. Salicylate toxicity model of tinnitus. Front Syst Neurosci (2012) 6:28. 10.3389/fnsys.2012.00028 22557950 PMC3341117

[113] Stolzberg D Hayes SH Kashanian N Radziwon K Salvi RJ Allman BL. A novel behavioral assay for the assessment of acute tinnitus in rats optimized for simultaneous recording of oscillatory neural activity. J Neurosci Methods (2013) 219:224–32. 10.1016/j.jneumeth.2013.07.021 23933328 PMC3796950

[114] Sheppard A Hayes SH Chen GD Ralli M Salvi R. Review of salicylate-induced hearing loss, neurotoxicity, tinnitus and neuropathophysiology. Acta Otorhinolaryngol Ital (2014) 34:79–93. 24843217 PMC4025186

[115] Sun W. Research on biological mechanisms of hyperacusis using animal models. ASHA Lead (2009) 14:5–6

[116] Sun W Lu J Stolzberg D Gray L Deng A Lobarinas E Salicylate increases the gain of the central auditory system. Neuroscience (2009) 159:325–34. 10.1016/j.neuroscience.2008.12.024 19154777 PMC2759817

[117] Zhang C Flowers E Li J-X Wang Q Sun W. Loudness perception affected by high doses of salicylate – a behavioral model of hyperacusis. Behav Brain Res (2014) 271:16–22. 10.1016/j.bbr.2014.05.045 24882611

[118] Wier CC Pasanen EG McFadden D. Partial dissociation of spontaneous otoacoustic emissions and distortion products during aspirin use in humans. J Acoust Soc Am (1988) 84:230–7. 10.1121/1.396970 3411052

[119] Kujawa SG Fallon M Bobbin RP. Intracochlear salicylate reduces low-intensity acoustic and cochlear microphonic distortion products. Hear Res (1992) 64:73–80. 10.1016/0378-5955(92)90169-N 1490903

[120] Ruel J Chabbert C Nouvian R Bendris R Eybalin M Leger CL Salicylate enables cochlear arachidonic-acid-sensitive NMDA receptor responses. J Neurosci (2008) 28:7313–23. 10.1523/JNEUROSCI.5335-07.2008 18632935 PMC6670386

[121] Ralli M Lobarinas E Fetoni AR Stolzberg D Paludetti G Salvi R. Comparison of salicylate- and quinine-induced tinnitus in rats: development, time course, and evaluation of audiologic correlates. Otol Neurotol (2010) 31:823–31. 10.1097/MAO.0b013e3181de4662 20502380 PMC2893285

[122] Stolzberg D Chen GD Allman BL Salvi RJ. Salicylate-induced peripheral auditory changes and tonotopic reorganization of auditory cortex. Neuroscience (2011) 180:157–64. 10.1016/j.neuroscience.2011.02.005 21310217 PMC3070811

[123] Oliver D He DZ Klocker N Ludwig J Schulte U Waldegger S Intracellular anions as the voltage sensor of prestin, the outer hair cell motor protein. Science (2001) 292:2340–3. 10.1126/science.1060939 11423665

[124] Liberman MC Gao JG He DZ Wu XD Jia SP Zuo J. Prestin is required for electromotility of the outer hair cell and for the cochlear amplifier. Nature (2002) 419:300–4. 10.1038/nature01059 12239568

[125] Müller M Klinke R Arnold W Oestreicher E. Auditory nerve fibre responses to salicylate revisited. Hear Res (2003) 183:37–43. 10.1016/S0378-5955(03)00217-X 13679136

[126] Drexl M Lagarde MM Zuo J Lukashkin AN Russell IJ. The role of prestin in the generation of electrically evoked otoacoustic emissions in mice. J Neurophysiol (2008) 99:1607–15. 10.1152/jn.01216.2007 18234980

[127] Lagarde MM Drexl M Lukashkin AN Zuo J Russell IJ. Prestin’s role in cochlear frequency tuning and transmission of mechanical responses to neural excitation. Curr Biol (2008) 18:200–2. 10.1016/j.cub.2008.01.006 18221877

[128] Jeanmonod D Magnin M Morel A. Low-threshold calcium spike bursts in the human thalamus – common physiopathology for sensory, motor and limbic positive symptoms. Brain (1996) 119:363–75. 10.1093/brain/119.2.363 8800933

[129] Mühlau M Rauschecker JP Oestreicher E Gaser C Röttinger M Wohlschläger AM Structural brain changes in tinnitus. Cereb Cortex (2006) 16:1283–8. 10.1093/cercor/bhj070 16280464

[130] Vanneste S Plazier M der Loo Ev de Heyning PV Congedo M De Ridder D. The neural correlates of tinnitus-related distress. Neuroimage (2010) 52:470–80. 10.1016/j.neuroimage.2010.04.029 20417285

[131] Stolzberg D Chrostowski M Salvi RJ Allman BL. Intracortical circuits amplify sound-evoked activity in primary auditory cortex following systemic injection of salicylate in the rat. J Neurophysiol (2012) 108:200–14. 10.1152/jn.00946.2011 22496535 PMC3434608

[132] Barth DS Di S. 3-Dimensional analysis of auditory evoked-potentials in the rat neocortex. J Neurophysiol (1990) 64:1527–36. 2283539 10.1152/jn.1990.64.5.1527

[133] Prieto JJ Winer JA. Layer VI in cat primary auditory cortex: Golgi study and sublaminar origins of projection neurons. JComp Neurol (1999) 404:332–58. 10.1002/(SICI)1096-9861(19990215)404:3<332::AID-CNE5>3.0.CO;2-R 9952352

[134] Winer JA Prieto JJ. Layer V in cat primary auditory cortex (AI): cellular architecture and identification of projection neurons. J Comp Neurol (2001) 434:379–412. 10.1002/cne.1183 11343289

[135] Szymanski FD Garcia-Lazaro JA Schnupp JW. Current source density profiles of stimulus-specific adaptation in rat auditory cortex. J Neurophysiol (2009) 102:1483–90. 10.1152/jn.00240.2009 19571199

[136] Szymanski FD Rabinowitz NC Magri C Panzeri S Schnupp JW. The laminar and temporal structure of stimulus information in the phase of field potentials of auditory cortex. J Neurosci (2011) 31:15787–801. 10.1523/JNEUROSCI.1416-11.2011 22049422 PMC6623019

[137] Stolzberg D Lu J Schlee W Weisz N Sun W Salvi R. Salicylate-induced tinnitus: spectral changes in spontaneous ensemble activity in auditory cortex of Awake Rats. ARO Midwinter Meeting (2008).

[138] Lu J Lobarinas E Deng A Goodey R Stolzberg D Salvi RJ GABAergic neural activity involved in salicylate-induced auditory cortex gain enhancement. Neuroscience (2011) 189:187–98. 10.1016/j.neuroscience.2011.04.073 21664433 PMC3153886

[139] Zhang X Yang P Cao Y Qin L Sato Y. Salicylate induced neural changes in the primary auditory cortex of awake cats. Neuroscience (2011) 172:232–45. 10.1016/j.neuroscience.2010.10.073 21044658

[140] Happel MF Jeschke M Ohl FW. Spectral integration in primary auditory cortex attributable to temporally precise convergence of thalamocortical and intracortical input. J Neurosci (2010) 30:11114–27. 10.1523/JNEUROSCI.0689-10.2010 20720119 PMC6633479

[141] Rizzardo R Savastano M Maron MB Mangialaio M Salvadori L. Psychological distress in patients with tinnitus. J Otolaryngol (1998) 27:21–5 9511115

[142] Andersson G Freijd A Baguley DM Idrizbegovic E. Tinnitus distress, anxiety, depression, and hearing problems among cochlear implant patients with tinnitus. J Am Acad Audiol (2009) 20:315–9. 10.3766/jaaa.20.5.5 19585962

[143] Cima RF Crombez G Vlaeyen JW. Catastrophizing and fear of tinnitus predict quality of life in patients with chronic tinnitus. Ear Hear (2011) 32:634–41. 10.1097/AUD.0b013e31821106dd 21399500

[144] Zald DH Pardo JV. The neural correlates of aversive auditory stimulation. Neuroimage (2002) 16:746–53. 10.1006/nimg.2002.1115 12169258

[145] Maudoux A Lefebvre P Cabay J-E Demertzi A Vanhaudenhuyse A Laureys S Auditory resting-state network connectivity in tinnitus: a functional MRI study. PLoS One (2012) 7(5):e36222. 10.1371/journal.pone.0036222 22574141 PMC3344851

[146] Maudoux A Lefebvre P Cabay JE Demertzi A Vanhaudenhuyse A Laureys S Connectivity graph analysis of the auditory resting state network in tinnitus. Brain Res (2012) 1485:10–21. 10.1016/j.brainres.2012.05.006 22579727

[147] Seydell-Greenwald A Leaver AM Turesky TK Morgan S Kim HJ Rauschecker JP. Functional MRI evidence for a role of ventral prefrontal cortex in tinnitus. Brain Res (2012) 1485:22–39. 10.1016/j.brainres.2012.08.052 22982009 PMC3489972

[148] Crippa A Lanting CP van Dijk P Roerdink JB. A diffusion tensor imaging study on the auditory system and tinnitus. Open Neuroimag J (2010) 4:16–25. 10.2174/1874440001004010016 20922048 PMC2948149

[149] Kim HS Wan X Mathers DA Puil E. Selective GABA-receptor actions of amobarbital on thalamic neurons. Br J Pharmacol (2004) 143:485–94. 10.1038/sj.bjp.0705974 15381635 PMC1575418

[150] De Ridder D Fransen H Francois O Sunaert S Kovacs S Van De Heyning P. Amygdalohippocampal involvement in tinnitus and auditory memory. Acta Otolaryngol (2006) 126:50–3. 10.1080/03655230600895580 17114143

[151] Brennan JF Jastreboff PJ. Generalization of conditioned suppresion during salicylate-induced phantom auditory-perception in rats. Acta Neurobiol Exp (1991) 51:15–27. 1759596

[152] Kizawa K Kitahara T Horii A Maekawa C Kuramasu T Kawashima T Behavioral assessment and identification of a molecular marker in a salicylate-induced tinnitus in rats. Neuroscience (2010) 165:1323–32. 10.1016/j.neuroscience.2009.11.048 19958810

[153] Hildebrandt H Hoffmann NA Illing R-B. Synaptic reorganization in the adult rat’s ventral cochlear nucleus following its total sensory deafferentation. PLoS One (2011) 6:e23686. 10.1371/journal.pone.0023686 21887295 PMC3161744

[154] Middleton JW Kiritani T Pedersen C Turner JG Shepherd GM Tzounopoulos T. Mice with behavioral evidence of tinnitus exhibit dorsal cochlear nucleus hyperactivity because of decreased GABAergic inhibition. Proc Natl Acad Sci U S A (2011) 108:7601–6. 10.1073/pnas.1100223108 21502491 PMC3088638

[155] Asako M Holt AG Griffith RD Buras ED Altschuler RA. Deafness-related decreases in glycine-immunoreactive labeling in the rat cochlear nucleus. J Neurosci Res (2005) 81:102–9. 10.1002/jnr.20542 15929063 PMC4455948

[156] Wang H Brozoski TJ Turner JG Ling L Parrish JL Hughes LF Plasticity at glycinergic synapses in dorsal cochlear nucleus of rats with behavioral evidence of tinnitus. Neuroscience (2009) 164:747–59. 10.1016/j.neuroscience.2009.08.026 19699270 PMC2761999

[157] Suneja SK Benson CG Potashner SJ. Glycine receptors in adult guinea pig brain stem auditory nuclei: regulation after unilateral cochlear ablation. Exp Neurol (1998) 154:473–88. 10.1006/exnr.1998.6946 9878183

[158] Suneja SK Potashner SJ Benson CG. Plastic changes in glycine and GABA release and uptake in adult brain stem auditory nuclei after unilateral middle ear ossicle removal and cochlear ablation. Exp Neurol (1998) 151:273–88. 10.1006/exnr.1998.6812 9628763

[159] Potashner SJ Suneja SK Benson CG. Altered glycinergic synaptic activities in guinea pig brain stem auditory nuclei after unilateral cochlear ablation. Hear Res (2000) 147:125–36. 10.1016/S0378-5955(00)00126-X 10962179

[160] Bledsoe SC Nagase S Miller JM Altschuler RA. Deafness-induced plasticity in the mature central auditory system. Neuroreport (1995) 7:225–9. 10.1097/00001756-199512290-00054 8742457

[161] Milbrandt JC Holder TM Wilson MC Salvi RJ Caspary DM. GAD levels and muscimol binding in rat inferior colliculus following acoustic trauma. Hear Res (2000) 147:251–60. 10.1016/S0378-5955(00)00135-0 10962189

[162] Wang H Brozoski TJ Caspary DM. Inhibitory neurotransmission in animal models of tinnitus: maladaptive plasticity. Hear Res (2011) 279:111–7. 10.1016/j.heares.2011.04.004 21527325 PMC3172385

[163] Dong S Rodger J Mulders WH Robertson D. Tonotopic changes in GABA receptor expression in guinea pig inferior colliculus after partial unilateral hearing loss. Brain Res (2010) 1342:24–32. 10.1016/j.brainres.2010.04.067 20438718

[164] Yang S Weiner BD Zhang LS Cho S-J Bao S. Homeostatic plasticity drives tinnitus perception in an animal model. Proc Natl Acad Sci U S A (2011) 108:14974–9. 10.1073/pnas.1107998108 21896771 PMC3169130

[165] Kotak VC Sanes DH. Synaptically-evoked prolonged depolarizations in the developing auditory-system. J Neurophysiol (1995) 74:1611–20. 8989397 10.1152/jn.1995.74.4.1611

[166] Vale C Sanes DH. Afferent regulation of inhibitory synaptic transmission in the developing auditory midbrain. J Neurosci (2000) 20:1912–21. 10684892 10.1523/JNEUROSCI.20-05-01912.2000 PMC6772929

[167] Kotak VC Takesian AE Sanes DH. Hearing loss prevents the maturation of GABAergic transmission in the auditory cortex. Cereb Cortex (2008) 18:2098–108. 10.1093/cercor/bhm233 18222937 PMC2517109

[168] Sarro EC Kotak VC Sanes DH Aoki C. Hearing loss alters the subcellular distribution of presynaptic GAD and postsynaptic GABA(A) receptors in the auditory cortex. Cereb Cortex (2008) 18:2855–67. 10.1093/cercor/bhn044 18403398 PMC2583158

[169] Wang H-T Luo B Zhou K-Q Xu T-L Chen L. Sodium salicylate reduces inhibitory postsynaptic currents in neurons of rat auditory cortex. Hear Res (2006) 215:77–83. 10.1016/j.heares.2006.03.004 16632286

[170] Su Y-Y Luo B Wang H-T Chen L. Differential effects of sodium salicylate on current-evoked firing of pyramidal neurons and fast-spiking interneurons in slices of rat auditory cortex. Hear Res (2009) 253:60–6. 10.1016/j.heares.2009.03.007 19306920

[171] Isaacson Jeffry S Scanziani M. How inhibition shapes cortical activity. Neuron (2011) 72:231–43. 10.1016/j.neuron.2011.09.027 22017986 PMC3236361

[172] Atallah Bassam V Bruns W Carandini M Scanziani M. Parvalbumin-expressing interneurons linearly transform cortical responses to visual stimuli. Neuron (2012) 73:159–70. 10.1016/j.neuron.2011.12.013 22243754 PMC3743079

[173] Wilson NR Runyan CA Wang FL Sur M. Division and subtraction by distinct cortical inhibitory networks in vivo. Nature (2012) 488:343–8. 10.1038/nature11347 22878717 PMC3653570

[174] Moore AK Wehr M. Parvalbumin-expressing inhibitory interneurons in auditory cortex are well-tuned for frequency. J Neurosci (2013) 33:13713–23. 10.1523/JNEUROSCI.0663-13.2013 23966693 PMC3755717

[175] Pi H-J Hangya B Kvitsiani D Sanders JI Huang ZJ Kepecs A. Cortical interneurons that specialize in disinhibitory control. Nature (2013) 503:521–4. 10.1038/nature12676 24097352 PMC4017628

[176] Potashner SJ Suneja SK Benson CG. Regulation of D-aspartate release and uptake in adult brain stem auditory nuclei after unilateral middle ear ossicle removal and cochlear ablation. Exp Neurol (1997) 148:222–35. 10.1006/exnr.1997.6641 9398464

[177] Godfrey DA Godfrey MA Ding D-L Chen K Salvi RJ. Amino acid concentrations in chinchilla cochlear nucleus at different times after carboplatin treatment. Hear Res (2005) 206:64–73. 10.1016/j.heares.2005.03.004 16080999

[178] Illing R-B Kraus KS Meidinger MA. Reconnecting neuronal networks in the auditory brainstem following unilateral deafening. Hear Res (2005) 206:185–99. 10.1016/j.heares.2005.01.016 16081008

[179] Godfrey DA Jin Y-M Liu X Godfrey MA. Effects of cochlear ablation on amino acid levels in the rat cochlear nucleus and superior olive. Hear Res (2014) 309:44–54. 10.1016/j.heares.2013.11.005 24291808 PMC5819880

[180] Dong S Mulders WH Rodger J Robertson D. Changes in neuronal activity and gene expression in guinea-pig auditory brainstem after unilateral partial hearing loss. Neuroscience (2009) 159:1164–74. 10.1016/j.neuroscience.2009.01.043 19356697

[181] Dong S Mulders WH Rodger J Woo S Robertson D. Acoustic trauma evokes hyperactivity and changes in gene expression in guinea-pig auditory brainstem. Eur J Neurosci (2010) 31:1616–28. 10.1111/j.1460-9568.2010.07183.x 20525074

[182] Foerster CR Illing RB. Redistribution of NMDA receptors in the cochlear nucleus following cochleotomy. Neuroreport (1998) 9:3531–5. 10.1097/00001756-199810260-00036 9855312

[183] Rubio ME. Redistribution of synaptic AMPA receptors at glutamatergic synapses in the dorsal cochlear nucleus as an early response to cochlear ablation in rats. Hear Res (2006) 21(6–217):154–67. 10.1016/j.heares.2006.03.007 16644159

[184] Whiting B Moiseff A Rubio ME. Cochlear nucleus neurons redistribute synaptic AMPA and glycine receptors in response to monaural conductive hearing loss. Neuroscience (2009) 163:1264–76. 10.1016/j.neuroscience.2009.07.049 19646510 PMC2760652

[185] Gulley RL Wenthold RJ Neises GR. Remodeling of neuronal membranes as an early response to deafferentation. A freeze-fracture study. J Cell Biol (1977) 75:837–50. 10.1083/jcb.75.3.837 925083 PMC2111593

[186] Redd EE Pongstaporn T Ryugo DK. The effects of congenital deafness on auditory nerve synapses and globular bushy cells in cats. Hear Res (2000) 147:160–74. 10.1016/S0378-5955(00)00129-5 10962182

[187] Vale C Sanes DH. The effect of bilateral deafness on excitatory and inhibitory synaptic strength in the inferior colliculus. Eur J Neurosci (2002) 16:2394–404. 10.1046/j.1460-9568.2002.02302.x 12492434

[188] Kotak VC Fujisawa S Lee FA Karthikeyan O Aoki C Sanes DH. Hearing loss raises excitability in the auditory cortex. J Neurosci (2005) 25:3908–18. 10.1523/JNEUROSCI.5169-04.2005 15829643 PMC1764814

[189] Suneja SK Potashner SJ Benson CG. AMPA receptor binding in adult guinea pig brain stem auditory nuclei after unilateral cochlear ablation. Exp Neurol (2000) 165:355–69. 10.1006/exnr.2000.7471 10993695

[190] Pilati N Ison MJ Barker M Mulheran M Large CH Forsythe ID Mechanisms contributing to central excitability changes during hearing loss. Proc Natl Acad Sci U S A (2012) 109:8292–7. 10.1073/pnas.1116981109 22566618 PMC3361412

[191] Li S Choi V Tzounopoulos T. Pathogenic plasticity of Kv7.2/3 channel activity is essential for the induction of tinnitus. Proc Natl Acad Sci U S A (2013) 110:9980–5. 10.1073/pnas.1302770110 23716673 PMC3683764

[192] Lobarinas E Dalby-Brown W Stolzberg D Mirza NR Allman BL Salvi R. Effects of the potassium ion channel modulators BMS-204352 maxipost and its R-enantiomer on salicylate-induced tinnitus in rats. Physiol Behav (2011) 104(5):873–9. 10.1016/j.physbeh.2011.05.022 21640740

[193] Yang S Su W Bao S. Long-term, but not transient, threshold shifts alter the morphology and increase the excitability of cortical pyramidal neurons. J Neurophysiol (2012) 108(6):1567–74 22723674 10.1152/jn.00371.2012 PMC3544952

[194] Calford MB Rajan R Irvine DR. Rapid changes in the frequency tuning of neurons in cat auditory cortex resulting from pure-tone-induced temporary threshold shift. Neuroscience (1993) 55:953–64. 10.1016/0306-4522(93)90310-C 8232905

[195] Rajan R. Receptor organ damage causes loss of cortical surround inhibition without topographic map plasticity. Nat Neurosci (1998) 1:138–43. 10.1038/388 10195129

[196] Fritz J Shamma S Elhilali M Klein D. Rapid task-related plasticity of spectrotemporal receptive fields in primary auditory cortex. Nat Neurosci (2003) 6:1216–23. 10.1038/nn1141 14583754

[197] Dean I Harper NS McAlpine D. Neural population coding of sound level adapts to stimulus statistics. Nat Neurosci (2005) 8:1684–9. 10.1038/nn1541 16286934

[198] Fritz J Elhilali M Shamma S. Active listening: task-dependent plasticity of spectrotemporal receptive fields in primary auditory cortex. Hear Res (2005) 206:159–76. 10.1016/j.heares.2005.01.015 16081006

[199] Fritz JB Elhilali M Shamma SA. Differential dynamic plasticity of a1 receptive fields during multiple spectral tasks. J Neurosci (2005) 25:7623–35. 10.1523/JNEUROSCI.1318-05.2005 16107649 PMC6725393

[200] Dean I Robinson BL Harper NS McAlpine D. Rapid neural adaptation to sound level statistics. J Neurosci (2008) 28:6430–8. 10.1523/JNEUROSCI.0470-08.2008 18562614 PMC6670892

[201] Yin P Mishkin M Sutter M Fritz JB. Early stages of melody processing: stimulus-sequence and task-dependent neuronal activity in monkey auditory cortical fields A1 and R. J Neurophysiol (2008) 100:3009–29. 10.1152/jn.00828.2007 18842950 PMC2604844

[202] Middleton JW Tzounopoulos T. Imaging the neural correlates of tinnitus: a comparison between animal models and human studies. Front Syst Neurosci (2012) 6:35. 10.3389/fnsys.2012.00035 22586378 PMC3343475

[203] Levay S Hubel DH Wiesel TN. Pattern of ocular dominance columns in macaque visual cortex revealed by a reduced silver stain. JComp Neurol (1975) 159:559–75. 10.1002/cne.901590408 1092736

[204] Hubel DH Wiesel TN Levay S. Plasticity of ocular dominance columns in monkey striate cortex. Philos Trans R Soc Lond B Biol Sci (1977) 278:377. 10.1098/rstb.1977.0050 19791

[205] Darian-Smith C Gilbert CD. Axonal sprouting accompanies functional reorganization in adult cat striate cortex. Nature (1994) 368:737–40. 10.1038/368737a0 8152484

[206] Feldman DE Brecht M. Map plasticity in somatosensory cortex. Science (2005) 310:810–5. 10.1126/science.1115807 16272113

[207] Feldman DE. Synaptic mechanisms for plasticity in neocortex. Annu Rev Neurosci (2009) 32:33–55. 10.1146/annurev.neuro.051508.135516 19400721 PMC3071739

[208] Syka J. Plastic changes in the central auditory system after hearing loss, restoration of function, and during learning. Physiol Rev (2002) 82:601–36. 12087130 10.1152/physrev.00002.2002

[209] Baccus SA. From a whisper to a roar: adaptation to the mean and variance of naturalistic sounds. Neuron (2006) 51:682–4. 10.1016/j.neuron.2006.09.007 16982414

[210] Talwar SK Gerstein GL. Auditory frequency discrimination in the white rat. Hear Res (1998) 126:135–50. 10.1016/S0378-5955(98)00162-2 9872142

[211] Singh NC Theunissen FE. Modulation spectra of natural sounds and ethological theories of auditory processing. J Acoust Soc Am (2003) 114:3394–411. 10.1121/1.1624067 14714819

[212] Carandini M Heeger DJ. Normalization as a canonical neural computation. Nat Rev Neurosci (2012) 13:51–62. 10.1038/nrn3136 22108672 PMC3273486

[213] Robinson BL McAlpine D. Gain control mechanisms in the auditory pathway. Curr Opin Neurobiol (2009) 19:402–7. 10.1016/j.conb.2009.07.006 19665367

[214] Rabinowitz NC Willmore Ben DB Schnupp Jan WH King Andrew J. Contrast gain control in auditory cortex. Neuron (2011) 70:1178–91. 10.1016/j.neuron.2011.04.030 21689603 PMC3133688

[215] Willmore BD Cooke JE King AJ. Hearing in noisy environments: noise invariance and contrast gain control. J Physiol (2014) 592(Pt 16):3371–81. 10.1113/jphysiol.2014.274886 24907308 PMC4229334

[216] Olsen SR Wilson RI. Lateral presynaptic inhibition mediates gain control in an olfactory circuit. Nature (2008) 452:956–60. 10.1038/nature06864 18344978 PMC2824883

[217] Olsen SR Bhandawat V Wilson RI. Divisive normalization in olfactory population codes. Neuron (2010) 66:287–99. 10.1016/j.neuron.2010.04.009 20435004 PMC2866644

[218] Katzner S Busse L Carandini M. GABAA inhibition controls response gain in visual cortex. J Neurosci (2011) 31:5931–41. 10.1523/JNEUROSCI.5753-10.2011 21508218 PMC3083851

[219] Wehr M Zador AM. Balanced inhibition underlies tuning and sharpens spike timing in auditory cortex. Nature (2003) 426:442–6. 10.1038/nature02116 14647382

[220] Zhang LI Tan AY Schreiner CE Merzenich MM. Topography and synaptic shaping of direction selectivity in primary auditory cortex. Nature (2003) 424:201–5. 10.1038/nature01796 12853959

[221] Tan AY Zhang LI Merzenich MM Schreiner CE. Tone-evoked excitatory and inhibitory synaptic conductances of primary auditory cortex neurons. J Neurophysiol (2004) 92:630–43. 10.1152/jn.01020.2003 14999047

[222] Wu GK Arbuckle R Liu BH Tao HW Zhang LI. Lateral sharpening of cortical frequency tuning by approximately balanced inhibition. Neuron (2008) 58:132–43. 10.1016/j.neuron.2008.01.035 18400169 PMC2447869

[223] Wu GK Tao HW Zhang LI. From elementary synaptic circuits to information processing in primary auditory cortex. Neurosci Biobehav Rev (2011) 35:2094–104. 10.1016/j.neubiorev.2011.05.004 21609731 PMC3184206

[224] Carcea I Froemke RC. Chapter 3 – cortical plasticity, excitatory-inhibitory balance, and sensory perception. In: Michael M Merzenich MN Thomas MV, editors. Progress in Brain Research. (Vol. 207). Elsevier (2013). p. 65–90 10.1016/B978-0-444-63327-9.00003-5 PMC4300113 24309251

[225] Froemke RC Carcea I Barker AJ Yuan K Seybold BA Martins AR Long-term modification of cortical synapses improves sensory perception. Nat Neurosci (2013) 16:79–88. 10.1038/nn.3274 23178974 PMC3711827

[226] Rasmusson DD Turnbull BG. Immediate effect of digit amputation on SI cortex in the raccoon – unmasking of inhibitory fields. Brain Res (1983) 288:368–70. 10.1016/0006-8993(83)90120-8 6661627

[227] Calford MB Tweedale R. Acute changes in the cutaneous receptive-fields in primary somatosensory cortex after digit denergation in adult flying fox. J Neurophysiol (1991) 65:178–87. 2016636 10.1152/jn.1991.65.2.178

[228] Schmid LM Rosa MG Calford MB. Retinal detachment induces massive immediate in visual-cortex. Neuroreport (1995) 6:1349–53. 10.1097/00001756-199506090-00030 7670002

[229] Gilbert CD Das A Ito M Kapadia M Westheimer G. Spatial integration and cortical dynamics. Proc Natl Acad Sci U S A (1996) 93:615–22. 10.1073/pnas.93.2.615 8570604 PMC40100

[230] Gilbert CD. Adult cortical dynamics. Physiol Rev (1998) 78:467–85. 9562036 10.1152/physrev.1998.78.2.467

[231] Scholl B Wehr M. Disruption of balanced cortical excitation and inhibition by acoustic trauma. J Neurophysiol (2008) 100(2):646–56. 10.1152/jn.90406.2008 18525018

[232] Salvi RJ Henderson D Fiorino F Colletti V. Auditory System Plasticity and Regeneration. New York, NY: Thieme Medical Publishers, Inc (1996).

[233] Caspary DM Backoff PM Finlayson PG Palombi PS. Inhibitory inputs modulate discharge rate within frequency receptive-fields of anteroventral cochlear nucleus neurons. J Neurophysiol (1994) 72:2124–33. 7884448 10.1152/jn.1994.72.5.2124

[234] Ehret G Merzenich MM. Complex sound analysis (frequency resolution, filtering and spectral integration) by single units of the inferior colliculus of the cat. Brain Res (1988) 472:139–63. 10.1016/0165-0173(88)90018-5 3289688

[235] Suga N Zhang YF Yan J. Sharpening of frequency tuning by inhibition in the thalamic auditory nucleus of the mustached bat. J Neurophysiol (1997) 77:2098–114. 9114258 10.1152/jn.1997.77.4.2098

[236] Davis KA Young ED. Pharmacological evidence of inhibitory and disinhibitory neuronal circuits in dorsal cochlear nucleus. J Neurophysiol (2000) 83:926–40. 10669505 10.1152/jn.2000.83.2.926

[237] Wang J Caspary D Salvi RJ. GABA-A antagonist causes dramatic expansion of tuning in primary auditory cortex. Neuroreport (2000) 11:1137–40. 10.1097/00001756-200004070-00045 10790896

[238] Wang J McFadden SL Caspary D Salvi R. Gamma-aminobutyric acid circuits shape response properties of auditory cortex neurons. Brain Res (2002) 944:219–31. 10.1016/S0006-8993(02)02926-8 12106684

[239] Pollak GD. The dominant role of inhibition in creating response selectivities for communication calls in the brainstem auditory system. Hear Res (2013) 305:86–101. 10.1016/j.heares.2013.03.001 23545427 PMC3778109

[240] Young ED Voigt HF. Response properties of type-II and type-III units in dorsal cochlear nucleus hearing research. Hear Res (1982) 6:153–69 7061349 10.1016/0378-5955(82)90051-x

[241] Caspary DM Pazara KE Kossl M Faingold CL. Strychnine alters the fusiform cell output from the dorsal cochlear nucleus. Brain Res (1987) 417:273–82. 10.1016/0006-8993(87)90452-5 3651816

[242] Alkhatib A Biebel UW Smolders JW. Reduction of inhibition in the inferior colliculus after inner hair cell loss. Neuroreport (2006) 17:1493–7. 10.1097/01.wnr.0000234754.11431.ee 16957595

[243] Xie R Gittleman JX Pollak GD. Rethinking tuning: in vivo whole-cell recordings of the inferior colliculus in awake bats. J Neurosci (2007) 27(35):9469–81. 10.1523/JNEUROSCI.2865-07.2007 17728460 PMC6673120

[244] Noreña AJ. An integrative model of tinnitus based on a central gain controlling neural sensitivity. Neurosci Biobehav Rev (2011) 35:1089–109. 10.1016/j.neubiorev.2010.11.003 21094182

[245] Smith EC Lewicki MS. Efficient auditory coding. Nature (2006) 439:978–82. 10.1038/nature04485 16495999

[246] Simoncelli EP Olshausen BA. Natural image statistics and neural representation. Annu Rev Neurosci (2001) 24:1193–216. 10.1146/annurev.neuro.24.1.1193 11520932

[247] Nelken I Rotman Y Yosef OB. Responses of auditory-cortex neurons to structural features of natural sounds. Nature (1999) 397:154–7. 10.1038/16456 9923676

[248] Chechik G Anderson MJ Bar-Yosef O Young ED Tishby N Nelken I. Reduction of information redundancy in the ascending auditory pathway. Neuron (2006) 51:359–68. 10.1016/j.neuron.2006.06.030 16880130

[249] Watkins PV Barbour DL. Specialized neuronal adaptation for preserving input sensitivity. Nat Neurosci (2008) 11:1259–61. 10.1038/nn.2201 18820690

[250] Turrigiano GG. Homeostatic plasticity in neuronal networks: the more things change, the more they stay the same. Trends Neurosci (1999) 22:221–7. 10.1016/S0166-2236(98)01341-1 10322495

[251] Schaette R Kempter R. Development of tinnitus-related neuronal hyperactivity through homeostatic plasticity after hearing loss: a computational model. Eur J Neurosci (2006) 23:3124–38. 10.1111/j.1460-9568.2006.04774.x 16820003

[252] Schaette R Kempter R. Development of hyperactivity after hearing loss in a computational model of the dorsal cochlear nucleus depends on neuron response type. Hear Res (2008) 240:57–72. 10.1016/j.heares.2008.02.006 18396381

[253] Schaette R Kempter R. Computational models of neurophysiological correlates of tinnitus. Front Syst Neurosci (2012) 6:34. 10.3389/fnsys.2012.00034 22586377 PMC3347476

[254] Schaette R McAlpine D. Tinnitus with a normal audiogram: physiological evidence for hidden hearing loss and computational model. J Neurosci (2011) 31:13452–7. 10.1523/JNEUROSCI.2156-11.2011 21940438 PMC6623281

[255] Franklin JL Fickbohm DJ Willard AL. Long-term regulation of neuronal calcium currents by prolonged changes of membrane-potential. J Neurosci (1992) 12:1726–35. 1315850 10.1523/JNEUROSCI.12-05-01726.1992 PMC6575878

[256] Turrigiano G Abbott LF Marder E. Activity-dependent changes in the intrinsic-properties of cultured neurons. Science (1994) 264:974–7. 10.1126/science.8178157 8178157

[257] Turrigiano GG. The self-tuning neuron: synaptic scaling of excitatory synapses. Cell (2008) 135:422–35. 10.1016/j.cell.2008.10.008 18984155 PMC2834419

[258] Turrigiano G. Homeostatic synaptic plasticity: local and global mechanisms for stabilizing neuronal function. Cold Spring Harb Perspect Biol (2012) 4(1):a005736. 10.1101/cshperspect.a005736 22086977 PMC3249629

[259] Pinchoff RJ Burkard RF Salvi RJ Coad ML Lockwood AH. Modulation of tinnitus by voluntary jaw movements. Am J Otol (1998) 19:785–9. 9831155

[260] Abel MD Levine RA. Muscle contractions and auditory perception in tinnitus patients and nonclinical subjects. Cranio (2004) 22:181–91. 10.1179/crn.2004.024 15293775

[261] Abraham WC Bear MF. Metaplasticity: the plasticity of synaptic plasticity. Trends Neurosci (1996) 19:126–30. 10.1016/S0166-2236(96)80018-X 8658594

[262] Koehler SD Shore SE. Stimulus timing-dependent plasticity in dorsal cochlear nucleus is altered in tinnitus. J Neurosci (2013) 33:19647–56. 10.1523/JNEUROSCI.2788-13.2013 24336728 PMC3858633

[263] Kotak VC Breithaupt AD Sanes DH. Developmental hearing loss eliminates long-term potentiation in the auditory cortex. Proc Natl Acad Sci U S A (2007) 104:3550–5. 10.1073/pnas.0607177104 17360680 PMC1805556

[264] Lambo ME Turrigiano GG. Synaptic and intrinsic homeostatic mechanisms cooperate to increase L2/3 pyramidal neuron excitability during a late phase of critical period plasticity. J Neurosci (2013) 33:8810–9. 10.1523/JNEUROSCI.4502-12.2013 23678123 PMC3700430

[265] Formby C Sherlock LP Gold SL. Adaptive plasticity of loudness induced by chronic attenuation and enhancement of the acoustic background. J Acoust Soc Am (2003) 114:55–8. 10.1121/1.1582860 12880017

[266] Plack CJ Carlyon RP Viemeister NF. Intensity discrimination under forward and backward-masking: role of referential coding. J Acoust Soc Am (1995) 97:1141–9. 10.1121/1.412227 7876436

[267] Roehl M Uppenkamp S. Neural coding of sound intensity and loudness in the human auditory system. J Assoc Res Otolaryngol (2012) 13:369–79. 10.1007/s10162-012-0315-6 22354617 PMC3346895

[268] Zeng FG. An active loudness model suggesting tinnitus as increased central noise and hyperacusis as increased nonlinear gain. Hear Res (2013) 295:172–9. 10.1016/j.heares.2012.05.009 22641191 PMC3593089

[269] Lockwood AH Salvi RJ Coad ML Towsley ML Wack DS Murphy BW. The functional neuroanatomy of tinnitus: evidence for limbic system links and neural plasticity. Neurology (1998) 50:114–20. 10.1212/WNL.50.1.114 9443467

[270] Lanting CP De Kleine E Bartels H Van Dijk P. Functional imaging of unilateral tinnitus using fMRI. Acta Otolaryngol (2008) 128:415–21. 10.1080/00016480701793743 18368576

[271] Melcher JR Levine RA Bergevin C Norris B. The auditory midbrain of people with tinnitus: abnormal sound-evoked activity revisited. Hear Res (2009) 257:63–74. 10.1016/j.heares.2009.08.005 19699287 PMC2760154

[272] Attias J Urbach D Gold S Shemesh Z. Auditory event related potentials in chronic tinnitus patients with noise induced hearing loss. Hear Res (1993) 71:106–13. 10.1016/0378-5955(93)90026-W 8113129

[273] Attias J Pratt H Haran IR Bresloff I Horowitz G Polyakov A Detailed analysis of auditory brainstem responses in patients with noise-induced tinnitus. Audiology (1996) 35:259–70. 10.3109/00206099609071946 8937658

[274] Gu J Herrmann B Levine R Melcher J. Brainstem auditory evoked potentials suggest a role for the ventral cochlear nucleus in tinnitus. J Assoc Res Otolaryngol (2012) 13:819–33. 10.1007/s10162-012-0344-1 22869301 PMC3505586

[275] Engineer ND Riley JR Seale JD Vrana WA Shetake JA Sudanagunta SP Reversing pathological neural activity using targeted plasticity. Nature (2011) 470:101–U114. 10.1038/nature09656 21228773 PMC3295231

[276] Westcott M. Acoustic shock injury (ASI). Acta Otolaryngol Suppl (2006) 126:54–8. 10.1080/03655230600895531 17114144

[277] Schecklmann M Landgrebe M Langguth B TRI Database Study Group. Phenotypic characteristics of hyperacusis in tinnitus. PLoS One (2014) 9:e86944. 10.1371/journal.pone.0086944 24498000 PMC3908961

[278] Dauman R Bouscau-Faure F. Assessment and amelioration of hyperacusis in tinnitus patients. Acta Otolaryngol (2005) 125:503–9. 16092541 10.1080/00016480510027565

[279] Goldstein B Shulman A. Tinnitus – hyperacusis and the loudness discomfort level test – a preliminary report. Int Tinnitus J (1996) 2:83–9. 10753346

[280] Hébert S Fournier P Noreña A. The auditory sensitivity is increased in tinnitus ears. J Neurosci (2013) 33:2356–64. 10.1523/JNEUROSCI.3461-12.2013 23392665 PMC6619157

[281] Ortmann M Müller N Schlee W Weisz N. Rapid increases of gamma power in the auditory cortex following noise trauma in humans. Eur J Neurosci (2011) 33:568–575 21198988 10.1111/j.1460-9568.2010.07542.x

[282] Fries P Reynolds JH Rorie AE Desimone R. Modulation of oscillatory neuronal synchronization by selective visual attention. Science (2001) 291:1560–3. 10.1126/science.1055465 11222864

[283] Fries P Nikolic D Singer W. The gamma cycle. Trends Neurosci (2007) 30:309–16. 10.1016/j.tins.2007.05.005 17555828

[284] Fries P Womelsdorf T Oostenveld R Desimone R. The effects of visual stimulation and selective visual attention on rhythmic neuronal synchronization in macaque area V4. J Neurosci (2008) 28:4823–35. 10.1523/JNEUROSCI.4499-07.2008 18448659 PMC3844818

[285] Tiesinga PH Sejnowski TJ. Mechanisms for phase shifting in cortical networks and their role in communication through coherence. Front Hum Neurosci (2010) 2(4):196. 10.3389/fnhum.2010.00196 21103013 PMC2987601

